# Stromal Ascorbate Peroxidase (*OsAPX7*) Modulates Drought Stress Tolerance in Rice (*Oryza sativa*)

**DOI:** 10.3390/antiox12020387

**Published:** 2023-02-05

**Authors:** Douglas Jardim-Messeder, Andreia Caverzan, Natalia Balbinott, Paloma K. Menguer, Ana L. S. Paiva, Moaciria Lemos, Juliana R. Cunha, Marcos L. Gaeta, Miguel Costa, Marcel Zamocky, Nelson J. M. Saibo, Joaquim A. G. Silveira, Rogério Margis, Márcia Margis-Pinheiro

**Affiliations:** 1 Departamento de Genética, Universidade Federal do Rio Grande do Sul, Porto Alegre 90010-150, RS, Brazil; 2 Instituto de Bioquímica Médica Leopoldo de Meis, Universidade Federal do Rio de Janeiro, Rio de Janeiro 21941-590, RJ, Brazil; 3 Departamento de Genética, Universidade Federal do Rio de Janeiro, Rio de Janeiro 21941-590, RJ, Brazil; 4 Centro de Biotecnologia, Universidade Federal do Rio Grande do Sul, Porto Alegre 90010-150, RS, Brazil; 5 Departamento de Bioquímica e Biologia Molecular, Universidade Federal do Ceará, Fortaleza 60020-181, CE, Brazil; 6 Departamento de Botânica, Universidade Federal Rio Grande do Sul, Porto Alegre 90010-150, RS, Brazil; 7 LEAF, TERRA, Instituto Superior de Agronomia, University of Lisbon, 1349-017 Lisboa, Portugal; 8 Laboratory of Phylogenomic Ecology, Institute of Molecular Biology, Slovak Academy of Sciences, Dúbravská cesta 21, 84551 Bratislava, Slovakia; 9 Department of Chemistry, Institute of Biochemistry, University of Natural Resources and Life Sciences, Vienna, Muthgasse 18, 1190 Vienna, Austria; 10 Instituto de Tecnologia Química e Biológica António Xavier, Universidade Nova de Lisboa, 2780-157 Oeiras, Portugal

**Keywords:** ascorbate peroxidase, reactive oxygen species, chloroplast, abiotic stress, drought, plant stress response, photosynthesis

## Abstract

Chloroplast ascorbate peroxidases exert an important role in the maintenance of hydrogen peroxide levels in chloroplasts by using ascorbate as the specific electron donor. In this work, we performed a functional study of the stromal APX in rice (OsAPX7) and demonstrated that silencing of OsAPX7 did not impact plant growth, redox state, or photosynthesis parameters. Nevertheless, when subjected to drought stress, silenced plants (APX7i) show a higher capacity to maintain stomata aperture and photosynthesis performance, resulting in a higher tolerance when compared to non-transformed plants. RNA-seq analyses indicate that the silencing of OsAPX7 did not lead to changes in the global expression of genes related to reactive oxygen species metabolism. In addition, the drought-mediated induction of several genes related to the proteasome pathway and the down-regulation of genes related to nitrogen and carotenoid metabolism was impaired in APX7i plants. During drought stress, APX7i showed an up-regulation of genes encoding flavonoid and tyrosine metabolism enzymes and a down-regulation of genes related to phytohormones signal transduction and nicotinate and nicotinamide metabolism. Our results demonstrate that OsAPX7 might be involved in signaling transduction pathways related to drought stress response, contributing to the understanding of the physiological role of chloroplast APX isoforms in rice.

## 1. Introduction

The reactive oxygen species (ROS) are produced from the monovalent reduction of molecular oxygen, being intrinsic to aerobic metabolism. In plants, ROS are produced mainly in response to adverse environmental conditions, acting as signaling molecules in different key physiological pathways related to development and stress response [1,2,3,4]. However, at high concentrations, ROS, such as hydrogen peroxide (H_2_O_2_), can diffuse through biological membranes and react with biological compounds such as lipids, proteins and nucleic acids, promoting damage and, in many cases, cellular death [5]. The control of intracellular ROS levels, and consequently, cellular redox homeostasis, is a complex process involving many mechanisms and a large gene network [6]. The main ROS scavenging mechanism is composed of several enzymes present in different subcellular compartments, such as superoxide dismutases (SOD), ascorbate peroxidases (APX), catalases (CAT), glutathione peroxidases (GPX), peroxiredoxins (Prx) and other heme and non-heme peroxidases [5,6,7].

APX (EC, 1.11.1.11) has an important role in H_2_O_2_ level maintenance in photosynthetic organisms, catalyzing the reduction of hydrogen peroxide using ascorbate as a specific electron donor [8]. APX is a component of the ascorbate-glutathione cycle, recognized as the main antioxidant system in plants. In this cycle, glutathione and NAD(P)H are used as substrates to restore the ascorbate levels [9,10]. In angiosperms, the APX family is composed of genes that encode cytosolic (cAPX), peroxisomal (pAPX), chloroplastic (chlAPX), and mitochondrial (mitAPX) isoforms [11].

In rice (*Oryza sativa* L.), APX is encoded by a small gene family, in which the isoforms are targeted to distinct subcellular compartments: cytosol (*OsAPX1* and *OsAPX2*), peroxisome (*OsAPX3* and *OsAPX4*), mitochondrial (*OsAPX5* and *OsAPX6*), chloroplast stroma (*OsAPX7*), and chloroplast thylakoid (*OsAPX8*) [12,13]. Rice plants with both chlAPXs simultaneously silenced exhibit differences in photosynthetic parameters related to the efficiency of light utilization and CO_2_ when subjected to stress conditions, suggesting that the reduced abundance of APX isoforms in chloroplast could modulate the photosystem activity and integrity [14]. Among the chlAPX isoforms, *OsAPX8* is repressed during drought stress in a mechanism related to increased ROS levels and stomata closure [14,15]. Indeed, plants exclusively silenced to *OsAPX8* display a higher H_2_O_2_ content and decreased stomata opening, and lower growth rate compared to non-transformed plants [15,16]. RNA-seq analysis showed that *OsAPX8* silencing mainly affects the expression of genes related to photosynthesis and signal transduction [15]. In addition, the overexpression of *OsAPX8* leads to increased tolerance to bacterial pathogens, while silenced lines are more sensitive than non-transformed plants [17]. These data suggest that the chlAPX isoforms exert important roles during the stress response; however, the individual contribution of *OsAPX7* in normal and stress conditions remains unknown.

The aim of this work is to perform a functional study of the *OsAPX7* gene using rice plants silenced by RNAi (APX7i) and to evaluate the drought stress response. Our results show that the transgenic plants displayed a growth similar to that of the non-transformed Nipponbare (Nipp) plants without changes in chloroplast APX activity, hydrogen peroxide content, or photosynthesis parameters under optimal growth conditions. However, when subjected to drought stress, APX7i plants showed a higher capacity to maintain stomata aperture and photosynthesis performance, resulting in a higher tolerance to drought stress, as compared to Nipp plants. RNA-seq analyses showed that APX7i plants subjected to drought stress displayed a similar gene expression profile to untreated plants, with fewer genes modulated in response to drought as compared to Nipp plants under stress conditions. Several drought-responsive genes were down-regulated in Nipp plants, while APX7i were able to maintain their expression levels, suggesting that the silencing of stromal OsAPX7 has a potential priming effect that enhances the capacity of plants to cope with water withhold.

## 2. Materials and Methods

### 2.1. Plant Material and Growth Conditions

Rice (*Oryza sativa* L. japonica cv. Nipponbare) seeds were germinated in MS medium (Sigma-Aldrich, St. Louis, MS, USA) at 25 °C with a 12-h photoperiod. One week after being sown, the rice seedlings were transferred to hydroponic growth in 200 mL plastic cups (three seedlings per cup) filled with Hoagland–Arnon’s nutritive solution [18]. ABA treatment consisted in spraying in excess an ABA solution (100 µM ABA dissolved in 0.1% Triton X-100) over the leaves of one-month-old plants until complete wetting was achieved. All experiments were conducted before the reproductive phase of rice plants.

### 2.2. Vector Construction and Plant Transformation 

A plasmid was constructed to express a hairpin structure (hpRNA) based on the sequence of the *OsAPX7* (*LOC_Os04g35520*) gene and was named OsAPX7i. The following primers were used to amplify a 220 bp fragment from the OsAPX7 gene to clone into the RNAi vector: 5′-CTCCGAGCAATCTGGGTGCAAAAAT-3′ and 5′-GGTACCTCGAGGACTCGTGGTCAGGAAAAGC-3′. The PCR product was cloned into the Gateway RNAi vector pANDA, in which the expression of the hairpin RNA is driven by the maize ubiquitin promoter [19]. Agrobacterium tumefaciens-mediated transformation was performed as previously described [20].

To make a translational fusion of the OsAPX7 protein with Yellow Fluorescent Protein (YFP) on the N-terminus and HA on the C-terminus, the OsAPX7 (LOC_Os04g35520) CDS was amplified from rice leaf cDNA using the primers 5′-ATGGCGGCCCAGCG-3′ and 5′-ACCGTCCAACGTGAATCC-3′ and cloned into the pART7-HA-YFP plasmid [21]. The gene expression is under the control of the 35S promoter of CaMV. The construct was then used to transform rice protoplasts. 

### 2.3. Isolation and Transformation of Protoplasts

Protoplast isolation and transformation were performed as previously described [22,23]. After transformation, protoplasts were incubated for 24–48 h in the dark at 28 °C before imaging. Fluorescence was monitored using an Olympus FluoView 1000 confocal laser scanning microscope (Olympus, Tokyo, Japan) equipped with a set of filters capable of distinguishing between green and yellow fluorescent proteins (GFP and YFP, respectively) and plastid autofluorescence. The images were captured with a high-sensitivity photomultiplier tube detector.

### 2.4. Analysis of the OsAPX7 Promoter Expression Pattern in Rice Plants

Leaves, roots, stems, and panicles of transgenic rice plants expressing the GUS gene under the control of the *OsAPX7* promoter (promAPX7-GUS) were sampled and analyzed using the X-Gluc histochemical assay (Fermentas^®^, Waltham, MA, USA), as previously described [24], with minor modifications. After GUS staining, the samples were clarified with graded ethanol series (30–70%) and analyzed in stereomicroscope and bright field microscopy. 

### 2.5. Drought Stress

One-week-old rice seedlings were transferred to soil in 10 L plastic pots. These pots were filled with a mixture of soil, vermiculite and peat (2:1:1) in a phytotron (12-h-light/12-h-dark cycles, 250–400 μmol m^−2^ s^−1^; 400 μmol m^−2^ s^−1^ at the top of the plants) at 28/24 °C and 70% relative humidity. Each pot had six plants, which were grown under flooded soil. The drought treatment was performed with one-month-old rice plants by withholding watering. The progressive drought stress was maintained until the relative water content in the soil reached 10% (which took approximately 8 days), estimated by differential weighing. A: The relative water content in the soil was determined by weighing the water content relative to the dry weight of the soil. The leaf relative water content (RWC) was calculated as follows: RWC = [(FW − DW)/(TW − DW)] × 100, where FW is the fresh weight, TW is the turgid weight measured after 6 h of saturation in deionized water at 4 °C in the dark and DW is the dry weight determined after 48 h in an oven at 75 °C. 

### 2.6. Methyl Viologen (MV) Treatment

One-month-old rice plants were used for methyl viologen (MV) treatment. Seedlings were grown as described above. The MV was dissolved in 0.1% Triton-X-100 at 50 μM and sprayed in excess on the leaves of the plants until complete wetting was achieved. This procedure was repeated twice a day. The first symptoms of toxicity (brown spots) appeared on the leaves after 24 h of treatment. The control plants were sprayed with 0.1% Triton-X-100 in the same way as the plants that underwent MV treatment.

### 2.7. Quantitative PCR (RT-qPCR)

Real-time PCR experiments were carried out using cDNA synthesized from total leaves RNA purified with TRIzol (Invitrogen^®^ Waltham, MA, USA). The samples were treated with DNase (Invitrogen^®^) to remove the eventual genomic DNA contamination, and complementary DNA (cDNA) was obtained using the SuperscriptTMII (Life Technologies^®^ Carlsbad, CA, USA) reverse transcriptase system and a 24-polyTV primer. After synthesis, cDNAs were diluted 10–100 times in sterile water for use in PCR reactions. RT-qPCR reactions were performed in an Applied Biosystems StepOne plus real-time PCR system (Applied Biosystems^®^ Waltham, MA, USA) using SYBR Green and target genes are listed in Supplementary Table S1. All reactions were repeated four times, and expression data analyses were performed after comparative quantification of the amplified products using the 2^−ΔΔCt^ method [25,26].

### 2.8. Purification of Isolated Chloroplasts

The purification of isolated chloroplasts was conducted, as demonstrated previously [27].

### 2.9. Quantitative Measurement of H_2_O_2_ and Chlorophyll Pigment

Measurements of H_2_O_2_ content were performed by extracting H_2_O_2_ from leaves using Ampliflu Red (Sigma-Aldrich) oxidation [28,29]. Fluorescence was monitored using a fluorometer at excitation and emission wavelengths of 563 nm and 587 nm, respectively. Calibration was performed by the addition of known quantities of H_2_O_2_.

For total chlorophyll, chlorophyll a and b, obtained after extraction in acetone 80%, were determined spectrophotometrically at 663 and 649 nm. The amount of pigment was calculated using the equations proposed previously [30]:

Chlorophyll-a (mg/L) = 12.25 × Abs663 − 2.79 × Abs647(1)

Chlorophyll-b (mg/L) = 21.50 × Abs647 − 5.10 × Abs663 (2)

For conducting these experiments, four leaves from each plant were analyzed, discarding the oldest and the newest leaf.

### 2.10. Measurement of Chlorophyll Pigment in Leaves by Co-Focal Microscopy

To analyze the chlorophyll content, the leaf segments were washed with double distilled water and analyzed by using an Olympus FluoView 1000 confocal laser scanning microscope (Olympus, Tokyo, Japan) by a filter to plastid autofluorescence. The images were captured with a high-sensitivity photomultiplier tube detector.

### 2.11. Cell Membrane Damage Determination

Electrolyte leakage (membrane damage) was measured as previously described [31], analyzing four leaves from each plant. Leaf slices were washed and placed in tubes containing 10 mL of deionized water. The flasks were incubated in a shaker for 12 h, and the electric conductivity in the medium (L1) was measured. Then, the medium was boiled (95 °C) for 60 min, and the electric conductivity (L2) was measured again. The relative membrane damage (MD) was estimated by MD = L1/L2 × 100.

### 2.12. Imaging of Rice Stomata

The stomata from the abaxial side of leaves were visualized by scanning electron microscopy and subsequently quantified according to their aperture: completely open, partially open and completely closed. Leaves of 30-day-old plants were detached at approximately 12:00 PM, and the samples were prepared as previously described [32].

### 2.13. Gas Exchange and Photochemical Parameters

The gas exchange measurements and the following photochemistry parameters associated with the efficiency of photosystem II were measured after 24 h of drought stress. The following PSII parameters were measured: ΔF/F’m, the actual quantum yield of photosystem II, Fv/Fm, the potential quantum yield of photosystem II, and ETR, the apparent electron transport rate. The relative excess energy at the photosystem II level was calculated as EXC = [(Fv/Fm) − (ΔFv/F’m)]/(Fv/Fm) [33,34]. All of the parameters were measured with an Infrared Gas Analyzer coupled with a leaf chamber fluorometer (Li-6400-XT, LI-COR, Lincoln, Dearborn, MI, USA) according to the manufacturer’s instructions. ETR was calculated as ETR = (ΔF/F’m× PPFD × 0.5 × 0.84), where 0.5 is the presumed fraction of the excitation energy distributed to PSII and 0.84 is the assumed fraction of light absorbed by the leaf. The photochemical quenching coefficient [qP = (F’m − Fs)/(F’m − F’o)] and the non-photochemical quenching coefficient [NPQ = (Fm − F’m)/F’m], where Fm and Fo are, respectively, maximum and minimum fluorescence of dark-adapted leaves; F’m and Fs are, respectively, maximum and steady-state fluorescence in the light-adapted state, and F’o is minimum fluorescence after far-red illumination of the previously light-exposed leaves. A saturating pulse of red light (0.8 s, 8000 μmol m^−2^ s^−1^) was utilized. Leaf gas exchange measurements were made using an Infrared Gas Analyzer (Li-6400-XT, LI-COR Biosciences Inc., Lincoln, Dearborn, MI, USA). The light was provided by a red/blue LED light source at a photon irradiance of 1000 μmol m^−2^ s^−1^. All leaf measurements were taken under ambient CO_2_ conditions (380 ppm) at a constant leaf temperature of 28 °C, relative humidity of 78%, and vapor pressure deficit (VPD) of 1.8 Pa. The gas exchange variables measured were net photosynthesis (Pn), transpiration rate (E), and intercellular CO_2_ concentration (Ci).

### 2.14. Thermal and RGB Imaging

Thermography was performed using a ThermaCam B20 camera (FLIR Systems) equipped with an uncooled 320 × 240 microbolometer matrix detector in the 7- to 13-μm wavelength, with a thermal sensitivity (noise-equivalent differential temperature) below 0.05 °C with emissivity set at 0.96–0.97. These subsequent experiments were carried out at the same time of day (3:00 p.m.) in a dedicated darkroom about 1–1.5 m distance from plants under low wind speed to ensure temperature contrast between the lines. Two trays, with a total of plants, were imaged at each time. Background temperature was determined by measuring the temperature of a crumpled sheet of aluminum foil in a similar position to the leaves of interest, with the emissivity set at 1.0.

Thermal images were analyzed with the imaging analysis software FLIR ThermaCAM Researcher (FLIR Systems, Inc., Wilsonville, OR, USA) by selecting a total of about 50 points over the imaged leaves per group of plants. We also recorded RGB images to support the analysis of thermal images.

### 2.15. Enzymatic Assays

Nipp and APX7i plants’ mature leaves (approximately 1g) were immersed in liquid nitrogen, finely ground to a powder with a mortar and pestle, and 2 mL of 100 mM K-phosphate buffer, pH 6.8, containing 0.1 mM EDTA and 2 mM ascorbate, were added to protein extraction. After centrifugation at 12,000× *g* for 15 min at 4 °C, the soluble protein content of the supernatant was quantified using the Bradford method [35] and, subsequently, used to evaluate APX enzymatic activity. The activity of ascorbate peroxidase (APX) was measured by following the ascorbate oxidation by the decrease in absorbance at 290 nm, as previously described [36].

### 2.16. RNA-Seq Library Preparation

Total RNA was extracted from leaf samples using the Direct-zol RNA kit (Zymo Research). RNA-seq libraries preparation and sequencing were performed with TruSeq Stranded mRNA Library Prep kit by Macrogen Inc. (Seul, Republic of Korea). Illumina Novaseq sequencing platform was used to produce twelve 100 bp paired-end libraries, with approximately 50 Mb reads per sample. RNA-seq raw reads quality was assessed with FastQC software [37]. Trim Galore! (https://www.bioinformatics.babraham.ac.uk/projects/trim_galore/, accessed on 21 September 2022) was used to remove adapter sequences and low-quality reads, keeping only reads with >Q30 scores and minimum lengths of 85 nucleotides for downstream analyses. High-quality reads were mapped against *Oryza sativa* MSU7.0 genome assembly using HISAT2 [38]. 

### 2.17. Differential Expression Analysis and Gene Ontology Enrichment

To explore plants’ response to water deficit at the transcript level, differential expression analysis was conducted using SARtools with DESeq2 [39], and P-values were adjusted with Benjamini and Hochberg’s correction to reduce false discovery rate [40]. Comparisons were carried out between and within genotypes (Nipp and APX7i) and treatments (drought and watered). In order to maximize the identification of genes involved in stress response, genes with *p* < 0.05 were defined as differentially expressed, with no threshold set for fold change (FC). Genes were defined as up- or down-regulated according to the following pairwise comparisons: Nipp under drought vs. Nipp untreated (Nipp_D–drought-responsive genes), APX7i untreated vs. Nipp untreated (APX7i–genes modulated as a result of *OsAPX7* silencing) and APX7i under drought vs. APX7i untreated (APX7i_D–drought-responsive genes in plants silenced to *OsAPX7*). Venn diagrams were constructed using genes that fit these criteria. Differentially expressed genes were subjected to Gene Ontology and KEGG pathways enrichment analysis using ShinyGO 0.76.1 [41], with an FDR cutoff of 0.05. 

### 2.18. Functional Protein Association Networks

The functional protein association network was created using the STRING database (https://string-db.org/, accessed on 7 December 2022) and analyzed using MEDUSA v1.0 [42] and VIACOMPLEX v1.0 software [43].

### 2.19. Statistical Analysis

Data were plotted with GraphPad PRISM 5.0 (GraphPad Software Inc., La Jolla, CA, USA) and analyzed by one-way ANOVA and a posteriori Tukey’s test. *P*-values of 0.05 were considered statistically significant.

## 3. Results

### 3.1. OsAPX7 Protein Is Specifically Localized in Chloroplast Stroma

**Figure 1:**
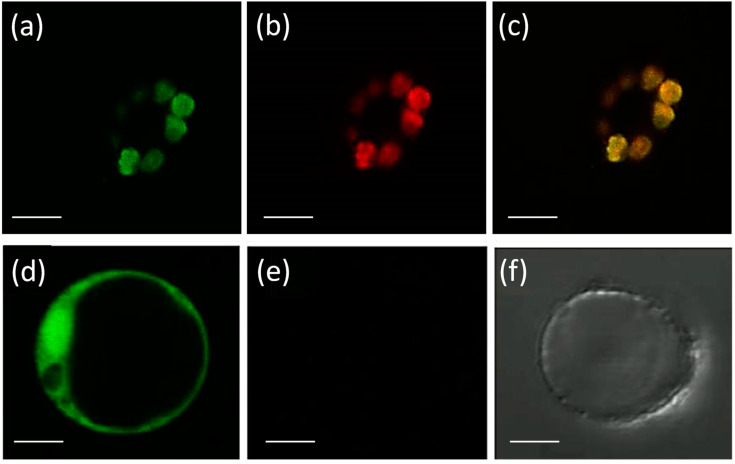
Subcellular localization of OsAPX7 protein in rice protoplasts. Subcellular localization of the OsAPX7 protein in chloroplasts in rice protoplasts through transient expression of the 35S-OsAPX7::YFP cassette. (**a**) green signals indicate YFP fluorescence; (**b**) red signals indicate chlorophyll autofluorescence, and (**c**) yellow signals are the merged image; (**d**) positive control of the localization, using 35S::YFP cassette; (**e**,**f**) negative control, using protoplast without 35S::YFP cassette. All scale bars represent 10 µm.

Eight APX genes are present in the rice genome [12,13], and the emergence of genes encoding stromal and thylakoidal exclusive isoforms (*OsAPX7*/*LOC_Os04g35520* and *OsAPX8*/*LOC_Os02g34810*, respectively) are the result of duplication and neofunctionalization events specific to Poaceae family [44]. To confirm the subcellular localization of stromal APX, we constructed a translational fusion of OsAPX7 with YFP protein driven by the CaMV 35S promoter. Confocal analysis of protoplasts expressing 35S-OsAPX7::YFP fusion confirmed that OsAPX7 localized specifically in chloroplast stroma (Figure 1).

### 3.2. Molecular and Morphological Analysis of OsAPX7 Silenced Plants

**Figure 2:**
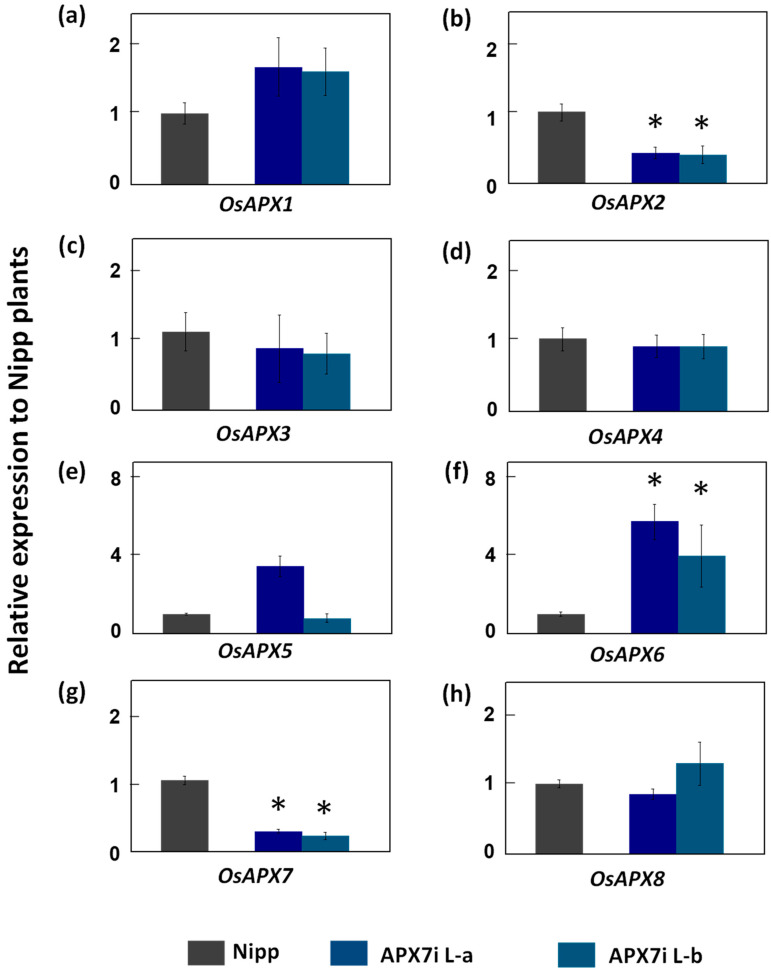
Gene expression analysis of ascorbate peroxidase family and other chloroplastic peroxidases in leaves of Nipp (Nipponbare) and APX7i plants. Genes analysed were (**a**) *OsAPX1*, (**b**) *OsAPX2*, (**c**) *OsAPX3*, (**d**) *OsAPX4*, (**e**) *OsAPX5*, (**f**) OsAPX6, (**g**) *OsAPX7*, (**h**) *OsAPX8*. The values were normalized by at least three constitutive genes and represent the average ± SE of relative expression to Nipp plants in at least three independent experiments. * indicates populations significantly different with *p* < 0.05.

To determine the functional role of stromal APX in rice plants, the *OsAPX7* gene was silenced using RNAi technology to generate transgenic lines named APX7i. The RT-qPCR analysis of APX7i T2 plants showed that *OsAPX7* transcript levels were down-regulated in two independent lines, named L-a and L-b (Figure 2). Among the cAPX isoforms, *OsAPX1* expression has no difference between Nipp and APX7i plants (Figure 2a). On the other hand, in both APX7i lines, the expression of *OsAPX2* was reduced in about 60% (Figure 2b). The silencing of OsAPX7 did not lead to changes in the expression of peroxisomal *OsAPX3* and *OsAPX4* (Figure 2c,d). The analysis of mitAPX shows that *OsAPX5* expression was not changed in APX7i lines (Figure 2e), while *OsAPX6* expression was increased (Figure 2f). Among the chlAPXs, the *OsAPX7* transcript was reduced by about 71% and 76% in lines L-a and L-b, respectively (Figure 2g), while the *OsAPX8* expression was not affected (Figure 2h).

To investigate the effect of *OsAPX7* silencing in plant development, the growth of APX7i plants was compared with Nipp plants. Our results demonstrated that, under optimal conditions, transgenic plants did not show apparent changes in growth when compared with non-transformed plants. Thus, all plants showed the same leaf length and growth rate (Supplementary Figure S1). The APX7i L-a was used as the representative line for the experiments described below.

### 3.3. Effect of OsAPX7 RNAi Silencing in Chloroplastic APX Activity and in Response to MV Treatment

**Figure 3:**
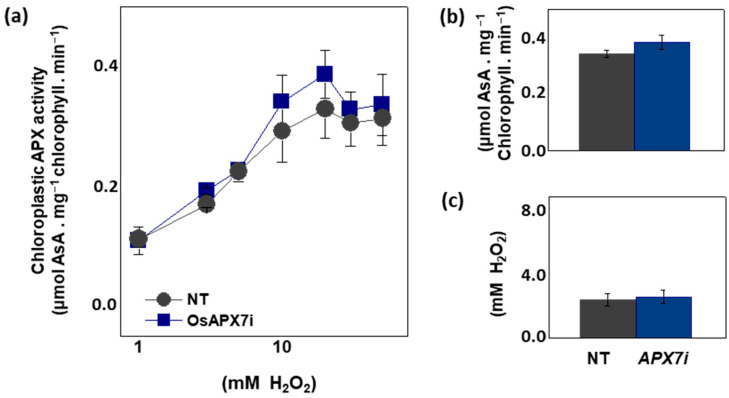
Chloroplastic APX activity and effect of MV treatment in chlorophyll content of Nipponbare (Nipp) and APX7i plants. (**a**) Measurement of APX activity under different H_2_O_2_ concentrations in isolated chloroplast from Nipp and APX7i plant leaves. (**b**) V_MAX_ of chloroplastic APX activity. (**c**) Apparent K_M_ of chloroplastic APX to H_2_O_2_.

To analyze the effect of *OsAPX7* silencing in chloroplastic APX activity, we measured the APX activity spectrophotometrically in isolated chloroplast from Nipp and APX7i plants. The APX activity was measured at 290 nm using different concentrations of H_2_O_2_. Our results demonstrate that *OsAPX7* silencing does not change the total chloroplastic APX activity (Figure 3a). In addition, we determined the catalytic parameters of the chloroplastic APX activity of Nipp and APX7i plants. The silencing of *OsAPX7* does not change the V_MAX_ value (Figure 3b) and the apparent K_M_ of H_2_O_2_ (Figure 3c).

**Figure 4:**
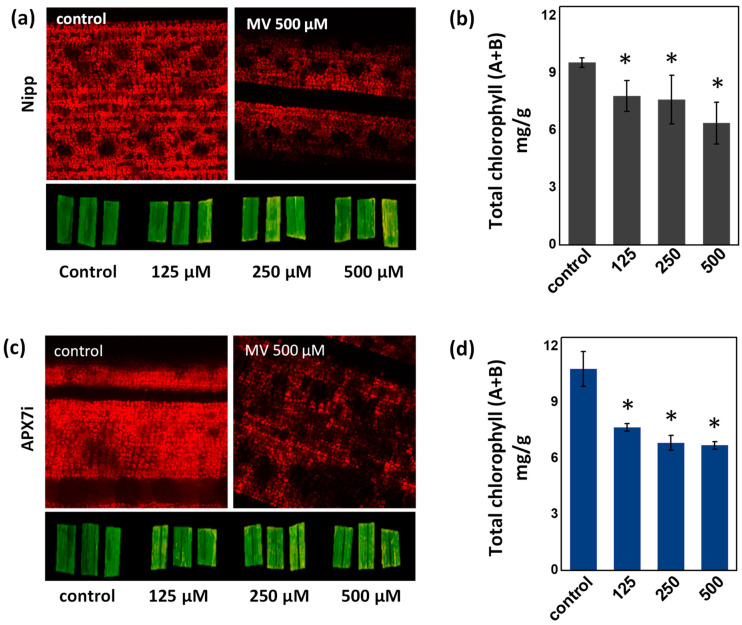
Effect of MV treatment in the chlorophyll content of Nipp plant leaves evaluated by fluorescence microscopy (**a**) and quantified by colorimetric method (**b**). Effect of MV treatment in the chlorophyll content of APX7i plant leaves evaluated by fluorescence microscopy (**c**) and quantified by colorimetric method (**d**). The values represent the average ± SE of at least four independent experiments. * indicates populations significantly different from the control situation with *p* < 0.05.

To determine the effect of stromal APX silencing in chloroplast stress response, we evaluated the change of chlorophyll content in leaves treated with methyl viologen (MV), which induces ROS production in the chloroplast. No differences in chlorophyll were observed under control conditions or in response to MV, demonstrating that the OsAPX7 silencing does not change the antioxidant capacity of chloroplasts (Figure 4a–d).

### 3.4. APX7i Plants Show Improved Drought Tolerance

**Figure 5:**
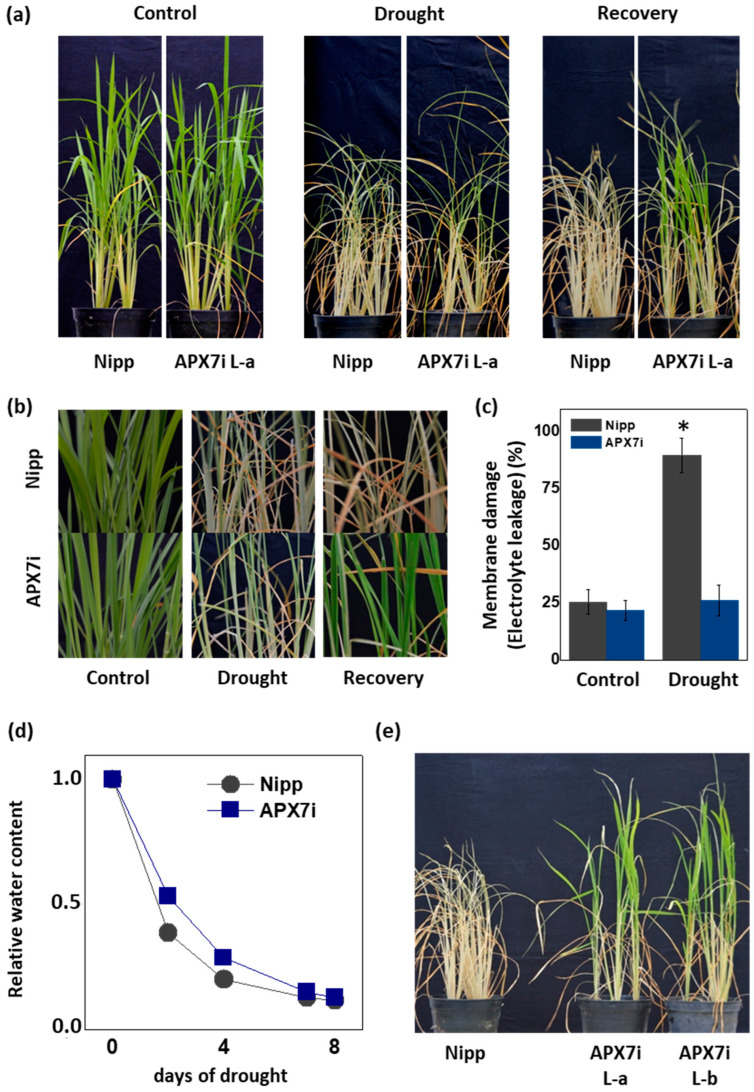
Effect of the OsAPX7 knockdown in drought stress tolerance. (**a**) Drought stress treatment in Nipponbare (Nipp) and APX7i plants. One-month-old plants (left panel) were subjected to eight days of drought (middle panel) and then recovered for three days (right panel). (**b**) Closer pictures of plants are shown in (**a**). (**c**) Membrane damage of Nipp (grey) and APX7i plants (blue) subjected to drought stress. (**d**) The relative water content of the soil in Nipp and APX7i plants subjected to drought stress. (**e**) Drought stress response in different lines of APX7i plants. One-month-old plants were subjected to eight days of drought and then recovered for three days. All values represent the average ± SE of at least four independent experiments. * indicates populations significantly different from the control situation with *p* < 0.05.

It has been demonstrated that the control of ROS production and their scavenging in the chloroplast is essential for drought tolerance [45,46,47,48]. To evaluate the effect of stromal APX silencing in drought stress response, we performed a soil drought experiment. Figure 5a,b show that APX7i plants have higher drought tolerance when compared to Nipp plants. To evaluate cell membrane damage in leaves of transgenic and NT plants subjected to drought stress, we determined the electrolyte leakage. Under drought stress, APX7i plants showed lower electrolyte leakage than Nipp plants (Figure 5c). The control of relative soil water content during the drought stress experiment is shown in Figure 5d, confirming that all lines were subjected to the same water deficit condition. In addition, we repeated the drought experiment using an additional line of OsAPX7i (L-b). Our results demonstrate that all APX7i lines show increased drought tolerance (Figure 5e), confirming that the silencing of *OsAPX7* improved drought tolerance and prevented cell membrane damage under drought stress.

**Figure 6:**
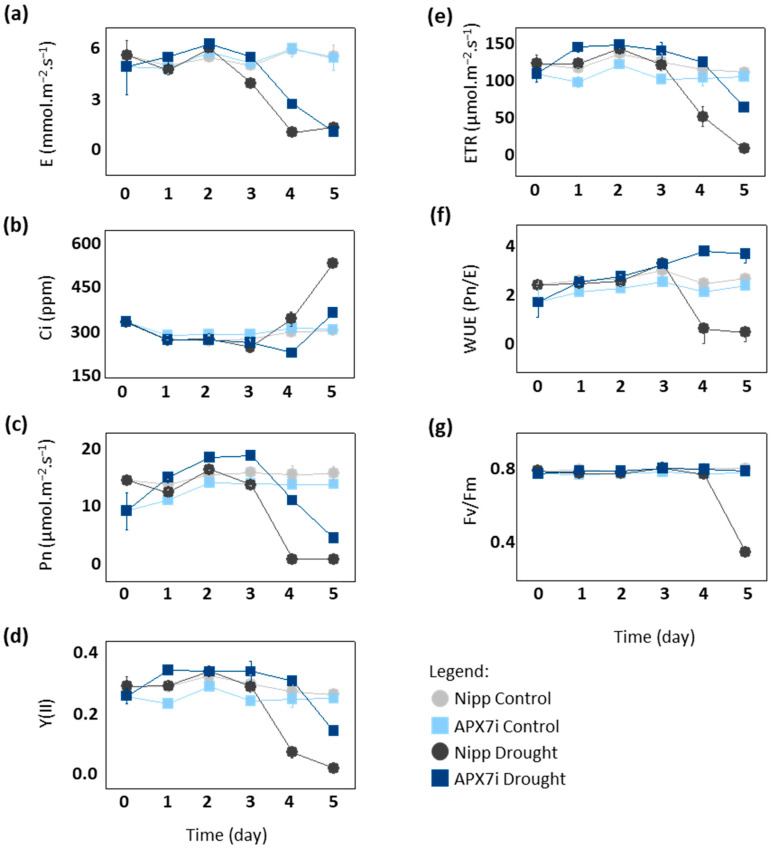
Photosynthetic and fluorescence parameters in plants subjected to drought stress. (**a**) transpiration, (**b**) intracellular CO_2_, (**c**) net photosynthesis, (**d**) quantum yield of photosystem II, (**e**) electron transport rate, (**f**) Water use efficiency, (**g**) Potential quantum use efficiency of photosynthesis (Fv/Fm) in Nipponbare (grey) and APX7i (blue) leaves. Light colors indicate the measurement in plants under control conditions. All values represent the media ± SE of at least three independent experiments.

To elucidate the physiological mechanism of drought tolerance in APX7i plants, we compared gas exchange and photochemical parameters between Nipp and APX7i under drought stress. After the fourth day of drought, Nipp plants showed a significant decrease in transpiration (E), net photosynthesis (Pn), photosystem II activity (YII) and electron transport rate (ETR), while APX7i plants maintained the values of these parameters slightly altered, being affected by drought stress only at the 5th day (Figure 6a,c,d,e, respectively). Consequently, APX7i plants showed higher internal CO_2_ concentrations than Nipp plants at a later stage of drought stress (Figure 6b). These results indicate that the enhanced drought tolerance of the APX7i plants might be due to an increased ability to maintain water use efficiency (WUE; Pn/unit of transpired water) associated with higher stability and activity of the quantum yield of photosystem II (Fv/Fm) under drought stress condition (Figure 6f,g, respectively).

### 3.5. OsAPX7 Silencing Leads to a Decrease in Stomata Opening

**Figure 7:**
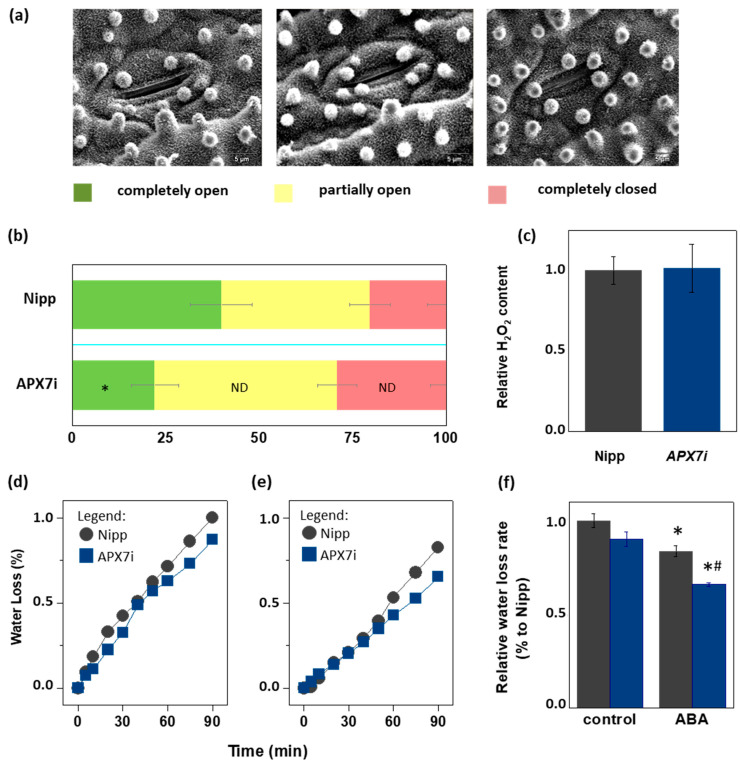
Effect of chloroplastic APX silencing in stomata opening and in leaf desiccation. (**a**) Environmental scanning electron microscopy images of three levels of stomatal opening. (**b**) Quantification of the percentage of the three levels of stomatal opening in leaves from Nipponbare (Nipp) and APX7i 30-day-old plants (*n* = 200 stomata in the abaxial side of four middle leaves for each line evaluated). * indicates populations significantly different from Nipp plants with *p* < 0.05, and ND indicates no difference. (**c**) The relative content of hydrogen peroxide in leaves from Nipp (grey) and APXi (blue) leaves. The values represent the average ± SE of at least five independent experiments. (**d**,**e**) Water loss of leaves detached from plants under control conditions (treated with water) and treated with ABA 100 μM, respectively. For each repeat, leaves of 30-day-old plants were used in a triplicate experiment (*n* = 3). The water loss rate of each condition was quantified in panel (**f**). Grey indicates Nipp, and blue indicates APX7i plants. * indicates populations significantly different from the control condition, and (#) indicates populations significantly different from Nipp plants with *p* < 0.05.

Due to the importance of stomata behavior during drought response, we compared the stomata aperture (Figure 7a) in Nipp and APX7i plants. Our results showed that Nipp plants have 20.3% of stomata completely closed, while in APX7i plants, this percentage increased to 29.0%. On the other hand, 39.9% of stomata were completely open in Nipp plants, while only 22.1% of stomata were completely open in APX7i plants. The percentage of partially open stomata was 39.7% and 48.8% in Nipp and APX7i plants, respectively, indicating no difference between the analyzed lines (Figure 7b). 

During the drought stress response, the stomata closure can be regulated by ROS metabolism [49]. In rice plants, drought stress leads to the down-regulation of the same antioxidant enzymes, being a mechanism proposed for the increased ROS amounts and stomata closure [32]. Indeed, during drought stress, thylakoidal *OsAPX8* is repressed, and the silencing of *OsAPX8* induces increased hydrogen peroxide content and stomata closure [15]. Here, we show that in APX7i plants, no difference was observed in the hydrogen peroxide content when compared to Nipp plants (Figure 7c). This result indicates that the increased stomata closure in APX7i plants is not related to a higher hydrogen peroxide content in leaves.

To determine the effect of *OsAPX7* silencing on leaf water loss, we also subjected rice leaves to a leaf water loss experiment. Our results demonstrated that *OsAPX7* silencing slightly decreased the leaf water loss (Figure 7d). Interestingly, this difference is more significant after pre-treatment with abscisic acid (ABA), which led to a reduced leaf water loss in both Nipp and APX7i plants (Figure 7e). The relative water loss rate is indicated in Figure 7f. Altogether, these results indicate that the silencing of *OsAPX7* leads to a decrease in stomata aperture accompanied by reduced leaf desiccation.

### 3.6. OsAPX7 Silencing Leads to a Decrease in Leaf Surface Temperature even under Drought Stress Conditions

**Figure 8:**
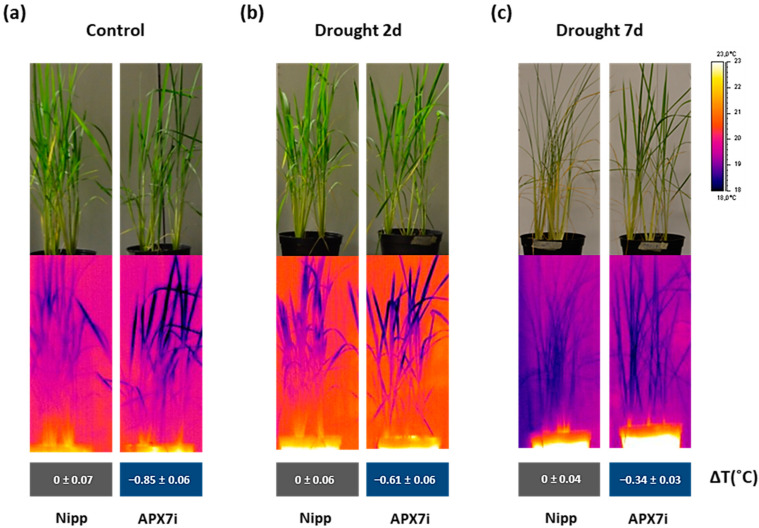
Thermal images of Nipponbare (Nipp) and APX7i captured by an infrared thermography device. (**a**) plants under control conditions, (**b**) plants under 2 days of drought stress, and (**c**) plants under 7 days of drought stress. The values represent the average ± SE of ΔT (°C) of each line to Nipp plants in three independent experiments.

An alternative approach for assessing stomata conductance is the infrared (IR) thermography in leaves. The leaf surface temperature correlates well with estimates of stomata conductance [50,51] and consequently with leaf water status [52]. Thus, we measured the leaf surface temperature in Nipp and APX7i plants under control conditions, moderate drought stress (2 days water withholding), and severe drought stress (7 days water withholding). Our results showed that under control conditions, the silencing of *OsAPX7* decreases leaf surface temperature by about 0.85 °C (Figure 8a). Under moderate and severe drought stress, the APX7i plants remain with a lower leaf surface temperature (−0.61 °C and −0.34 °C, respectively) (Figure 8b,c). These results indicate that although the silencing of *OsAPX7* led to increased stomata closure, the APX7i plants can maintain a lower leaf surface temperature, even under drought-stress conditions.

### 3.7. Effect of OsAPX7 Silencing on the Transcriptional Profile under Drought Stress

To explore the mechanisms behind APX7i plants’ higher drought tolerance, we investigated the transcriptional profiles of Nipp and APX7i plants under well-watered and drought conditions. A principal component analysis (PCA) produced four well-defined clusters corresponding to the four groups compared by our global transcriptomic and differential gene expression analyses (Supplementary Figure S2). Untreated APX7i and Nipp plants overlap along the first principal component (PC1), showing that genotype is not the main source of variability in the experiment. APX7i under drought stress is closer to untreated plants than Nipp plants under drought stress, showing that there is less variance between APX7i under drought and untreated plants than Nipp subjected to drought. 

**Figure 9:**
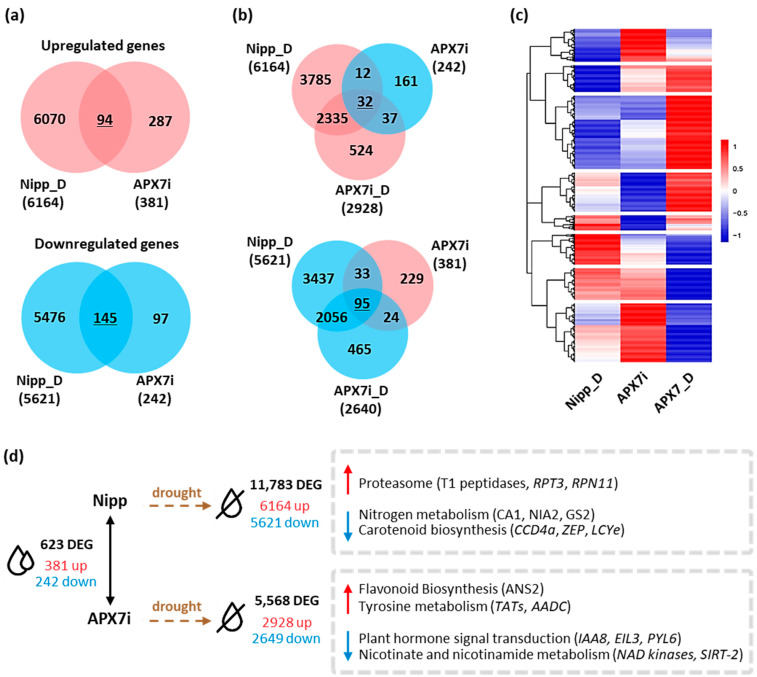
Differentially expressed genes in Nipponbare (Nipp) and APX7i rice plants under drought conditions. (**a**) Venn diagrams representing shared up- and down-regulated genes in Nipp plants under drought conditions (Nipp_D) and compared to untreated APX7i plants. Red circles represent up-regulated genes, and blue circles represent down-regulated genes. (**b**) Venn diagrams represent genes up- and down-regulated in both Nipp and APX7i plants under drought conditions (Nipp_D and APX7i_D) that display an inverse regulation profile in untreated APX7i plants. (**c**) Heatmap of 1462 differentially expressed genes, consisting of the intersections displayed in A and B, along with other induced and repressed genes in APX7i plants under drought conditions that are not differentially expressed in untreated APX7i plants. Red and blue shades indicate up- and down-regulated genes, respectively. (**d**) Differentially expressed genes between APX7i and Nipp rice plants and KEGG pathways enriched under drought treatment.

Pairwise comparisons between Nipp and APX7i plants under well-watered conditions revealed that the silencing of *OsAPX7* modulated the expression of 623 genes, with an increase in the expression of 381 and a reduction of 242 genes (Figure 9a,d). A stronger effect was observed when APX7i plants were subjected to drought stress and their profile compared to Nipp plants under similar treatment. Nipp plants subjected to 8 days of drought resulted in 11,783 differentially expressed genes (DEG), of which 6164 genes were up-regulated and 5621 were down-regulated. APX7i plants showed less than half of the number of modulated genes (5568) compared to Nipp plants, with less DEG in terms of repressed (2649) and induced (2928) genes. So far, it indicates a minor effect of drought stress in APX7i plants than in Nipp rice. Interestingly, untreated APX7i plants present a set of 127 DEG with a contrasting modulation pattern to plants under drought conditions (Figure 9b,c). A heatmap was built to represent the distinct expression profiles of the DEG, including contrasting expression along with the same up-regulated or down-regulated transcript profile in plants under drought stress (underlined numbers in Figure 9a,b).

A more specific and detailed analysis of the DEG can be made in terms of enriched metabolic pathways of KEGG (Figure 9d). We observed a 2.7-fold enrichment of genes related to the proteasome degradation pathway in Nipp plants under drought stress, including genes coding for the T1 endopeptidase 20S alpha1 subunit, 19S regulatory particle triple-A ATPase subunit 3 (RPT3) and 19S regulatory particle triple-A ATPase subunit 3 (RPN11), all with small or no induction in APX7i plants (Supplementary Figure S3a). Conversely, genes related to nitrogen metabolism and carotenoid biosynthesis were also enriched among the genes repressed in Nipp plants under drought, with a minor response in APX7i plants. This was observed and typified by six genes related to abiotic stresses: carotenoid-cleavage dioxygenase (CCD4), zeaxanthin epoxidase (ZEP), lycopene epsilon-cyclase (LCYe), beta-subunit of the carbonic anhydrase (CA1), nitrate reductase (NIA2) and chloroplastic glutamine synthase-2 (GS2) (Supplementary Figure S3b). Among the up-regulated DEG of APX7i plants under drought, genes related to flavonoid biosynthesis and tyrosine metabolism were enriched, whereas enriched KEGG pathways among the down-regulated genes include plant hormone signaling, and nicotinate and nicotinamide metabolism (Figure 9d and Supplementary Figure S4).

**Figure 10:**
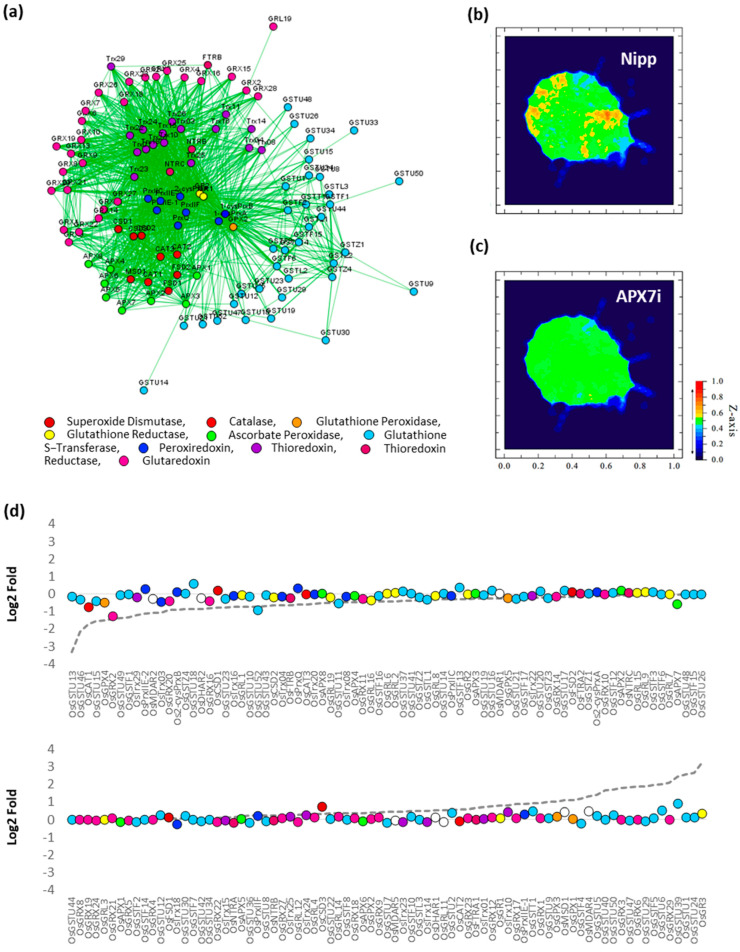
Effect of *OsAPX7* silencing on rice antioxidant network expression in response to drought. (**a**) Antioxidant network with different families relates to antioxidant metabolism. (**b**) Heatmap demonstrating the antioxidant network expression in Nipponbare (Nipp) during drought response. (**c**) Heatmap demonstrating the antioxidant network expression in APX7i during drought response. (**d**) Relative expression of antioxidant genes in Nipp and APX7i plants in response to drought. The circles indicate gene expression in APX7i plants. The colors are in accordance with the Figure 10a legend, and white circles indicate ascorbate reductase enzymes, which are not present in the network. The dotted line indicates the gene expression in Nipp plants.

To verify the effect of stromal APX silencing in rice antioxidant response, the transcription profile of an association network of different antioxidant enzymes was analyzed in response to drought by comparing Nipp and APX7i plants (Figure 10a). In Nipp plants, drought stress led to antioxidant response through the regulation of the expression of several genes related to ROS metabolism (Figure 10b,d). However, in APX7i plants, this response to drought was impaired, with the regulation of a few genes (Figure 10c,d). The individual expression of all genes of antioxidant metabolism used in the network in Nipp and APX7i plants in response to drought is shown in Supplementary Figure S5. In addition, the silencing of *OsAPX7* does not lead to changes in the expression of these genes in control conditions, confirming that the high tolerance to drought cannot be explained by changes in the antioxidant metabolism.

## 4. Discussion

The subcellular localization of all *OsAPX* family members was previously predicted by in silico analysis [12,13]. In chloroplast, chlAPX isoforms can be targeted to the stroma or thylakoid membrane. Although in most angiosperms, a unique gene encodes both mitAPX and chlAPX isoforms soluble in the stroma (sAPX) or bound to thylakoid membrane (tAPX), in Poales order, specific duplication and neofunctionalization events allow the emergence of individual genes encoding mitAPX, sAPX, and tAPX isoforms [44]. In the present study, our data confirmed the predicted subcellular localization of the *OsAPX7* protein, which is the stromal APX isoform. We previously demonstrated that the double silencing of both chloroplastic APXs (*OsAPX7* and *OsAPX8*) causes alterations in the level of proteins involved in photosynthesis and oxidative metabolism [14], while plants exclusively silenced to *OsAPX8* display a higher hydrogen peroxide content and decreased stomata opening and growth rate compared to Nipp plants [15]. Although the importance of rice chloroplastic APX isoforms in drought stress response, the role of stromal isoforms remained unknown. Here we conducted a functional study of the *OsAPX7* gene, showing its importance in ROS metabolism and drought stress response through the analysis of APX7i plants, specifically silenced for the gene *OsAPX7*.

Our results indicate that, in control conditions, the *OsAPX7* silencing does not impair chloroplastic APX activity and the normal development of rice plants. In addition, these plants do not exhibit symptoms of oxidative stress, such as membrane damage or reduced growth, and show a similar response to MV treatment compared to non-transformed Nipp plants. These data indicate that the silencing of stromal APX does not change the chloroplast antioxidant defense, and the expression data of APX7i plants discarded a possible compensatory transcriptional response of other enzymes related to ROS metabolism in the chloroplast. Thus, it is likely that the partial silencing of the *OsAPX7* does not significantly impact the control of H_2_O_2_ levels in the chloroplast.

**Figure 11:**
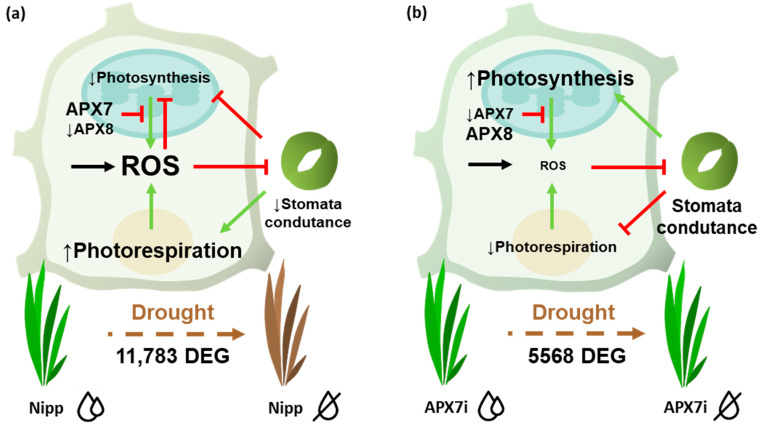
Schematic representation of pathways affected by APX7 silencing that can contribute to priming signaling and stress tolerance. (**a**) Drought stress response in Nipp plants. During the drought stress response, there is a reprogramming of gene expression and metabolism, leading to increased ROS production. In chloroplast, the thylakoidal *OsAPX8* is repressed, contributing to increasing ROS derived from photosynthesis. The high amounts of ROS inhibit photosynthesis and the stomata conductance, limiting gas exchange and CO_2_ uptake. This condition contributes to photosynthesis inhibition and to ROS production derived from photorespiration. (**b**) Drought stress response in APX7i. The silencing of *OsAPX7* impairs the transcription response. In chloroplast, *OsAPX8* is not repressed, and the ROS production is limited, maintaining the stomata conductance. The CO_2_ supply ensures the photosynthetic process and prevents photorespiration. Consequently, in APX7i plants, the ROS production and oxidative damage during drought stress are limited, contributing to the increased tolerance to stress.

ROS metabolism is directly involved with different stomata closure mechanisms and drought stress tolerance [47,49]. Here, we verified that despite the silencing of stromal APX does not change the cellular hydrogen peroxide content, and it led to a slight increase in stomata closure and increased tolerance to drought stress. The analysis of photochemical parameters and gas exchange in plants exposed to drought reveals that APX7i plants can maintain transpiration and, consequently, stomata conductance for a longer period under drought stress. These data suggest that the silencing of *OsAPX7* might activate the signaling pathway independent of a general increase of ROS levels in the chloroplast, maintaining the stomata conductance and preventing oxidative damage during drought stress (Figure 11).

The capacity to maintain the stomata conductance under stress conditions enables the maintenance of photosynthesis, resulting in a later increase in intracellular CO_2_ concentration. Although APX7i plants show a higher stomata closure than Nipp plants, this difference is not enough to decrease stomata conductance and transpiration, as verified previously in plants silenced to APX8 [15]. Consequently, APX7i plants can maintain the water use efficiency and the potential quantum yield of photosystem II for longer under drought-stress conditions. This later effect of drought stress on photosynthesis suggests that the silencing of *OsAPX7* prevents the inhibition of Calvin cycle biochemical reactions due to the ability of transformed plants to maintain the stomata conductance and the CO_2_ assimilation, even under drought stress. The maintenance of the Calvin cycle, therefore, enables continuous oxidation of NADPH to NADP^+^, which is required as the final electron acceptor of photosynthetic electron transporter chain activity, thus reducing the possibility of ROS generation [53].

The analysis of rice water status by leaf surface temperature demonstrated that the APX7i showed a lower temperature than Nipp plants, which remains low even under moderate or severe drought stress, confirming the high stomatal conductance. Indeed, lines that are more productive and tolerant to drought stress conditions, especially in crops, have been selected based on their lower leaf temperature, as demonstrated in wheat and maize [50].

Although the higher stomatal conductance allows greater capacities of plants to continue to perform photosynthesis and be potentially more productive, this strategy can be dangerous. A higher stomatal conductance under stressful conditions enables higher photosynthesis, but water loss by transpiration is also increased. This can be detrimental in severe and prolonged drought conditions, which could compromise not only production but also plant survival. 

At the transcriptional level, it became evident that the silencing of stromal APX triggered a series of downstream effects, with the establishment of a new transcriptional homeostatic state both at well-watered conditions and under drought stress. It is important to note that the number of genes modulated by drought in APX7i plants was less than half of the 11,783 genes observed in Nipp plants. This indicates that the silencing of *OsAPX7* promoted a potential priming effect that rendered plants more tolerant to drought stress. This response cannot be attributed to a small set of genes or a specific pathway, but some genes deserve particular attention as they are known to be involved in tolerance to abiotic stresses.

The modulation and reorganization of proteasome subunits are involved in developmental and stress-response processes [54,55]. Nipp plants under drought stress presented enrichment in the KEGG proteasome pathway, with 38 out of 58 genes up-regulated. To face drought and to increase protein turnover, Nipp plants responded with an up-regulation of T1 peptidases and the regulatory subunits RPN11 and RPT3. Most of the genes related to the proteasome remained near or at basal levels in APX7i plants under the same stress. It was previously demonstrated that knockout mutants of these genes increased plant abiotic stress susceptibility [56]. These data suggest that a basal level is required for proteostasis, but their overexpression can become more detrimental than beneficial for the plant.

Genes related to nitrogen metabolism were overrepresented among down-regulated genes in Nipp plants under drought. The chloroplast glutamine synthase overexpression in rice is associated with enhanced salt tolerance [57], and nitrate reductase [58] and carbonic anhydrase [59] have been directly implicated in plant tolerance to abiotic stresses. Several genes often associated with abiotic stress response were down-regulated both in Nipp and APX7i plants, but the reduction of transcript levels in APX7i plants was mitigated. A priming effect can be attributed to APX7, as silenced plants partially or completely rescued the down-regulation of these genes under stress. This pattern was observed for several other genes associated with nicotinamide and tyrosine metabolism, such as NAD kinase [60] and tyrosine aminotransferase [61], as well as genes involved in hormone signal transduction, such as PYL6 [62]. The ability of APX7i plants to maintain high expression levels of positive regulators of drought tolerance may be associated with the ability of APX7i plants to cope with water withholding and prevent oxidative stress, evidenced by increased membrane damage. On the other hand, oxidative stress, as a consequence of drought stress, activates the response of different genes of antioxidant metabolism in order to restore de-redox homeostasis.

This work reports for the first time the functional characterization of *OsAPX7* and that its silencing does not change the ROS metabolism and stomatal conductance under control conditions. On the other hand, the silencing of OsAPX7 led to plants with a major stomata conductance, lower leaf surface temperature and oxidative damage under drought stress, resulting in plants being more tolerant. More than a biotechnological application, this work reveals the complexity of the control exerted by the stromal APX in signaling transduction pathways regulating the abiotic stress responses. 

## Data Availability

All data presented in this study are available on request from the corresponding author.

## Supplementary Materials

The following supporting information can be downloaded at: https://www.mdpi.com/article/10.3390/antiox12020387/s1, Figure S1: Growth characterization of Nipponbare (Nipp) and APX7i plants; Figure S2: Principal component analysis of the four biological groups representing the non-transgenic plants (Nipp_C), OsAPX7 silenced plants (APX7i_C) and both groups when subjected to drought (Nipp_D and APX7i_D); Figure S3: Expression profile of genes differently expressed in non-transformed (Nipp) plants under drought stress; Figure S4: Expression profile of genes differently expressed in APX7i plants under drought stress; Figure S5: Gene expression analyses of antioxidant enzymes in rice; Table S1: Down-regulated genes in Nipp and APX7i plants under watered and drought conditions; Table S2: Up-regulated genes in Nipp and APX7i plants under watered and drought conditions; Table S3: DEGs resulting from multiple comparisons of gene expression profiles between Nipp and APX7i plants under watered and drought conditions.

## References

[1] Dat J., Vandenabeele S., Vranova E., Van Montagu M., Inze D., Van Breusegem F. (2000). Dual action of the active oxygen species during plant stress response. Cell Mol. Life Sci..

[2] Orozco-Cardenas M.L., Narvaez-Vasquez J., Ryan C.A. (2001). Hydrogen peroxide acts as a second messenger for the induction of defense genes in tomato plants in response to wounding, systemin, and methyl-jasmonate. Plant Cell.

[3] Mullineaux P., Karpinski S. (2002). Signal transduction in response to excess light: Getting out of the chloroplast. Curr. Opin. Plant Biol..

[4] Vandenabeele S., Van Der Kelen K., Dat J., Gadjev I., Boonefaes T., Morsa S., Rottiers P., Slooten L., Van Montagu M., Zabeau M. (2003). A comprehensive analysis of hydrogen peroxide-induced gene expression in tobacco. Proc. Natl. Acad. Sci. USA.

[5] Scandalios J.G. (2002). The rise of ROS. Trends Biochem Sci.

[6] Mittler R., Vanderauwera S., Gallery M., Van Breusegem F. (2004). Reactive oxygen gene network of plants. Trends Plant Sci..

[7] Chovanová K., Böhmer M., Poljovka A., Budiš J., Harichová J., Szemeš T., Zámocký M. (2020). Parallel Molecular Evolution of Catalases and Superoxide Dismutases Focus on Thermophilic Fungal Genomes. Antioxidants.

[8] Asada K. (1999). The water-water cycle in chloroplasts: Scavenging of active oxygen and dissipation of excess photons. Annu. Rev. Plant Physiol. Plant Mol. Biol..

[9] Alscher R.G., Donahue J.L., Cramer C.L. (1997). Reactive oxygen species and antioxidants: Relationships in green cells. Physiol. Plant.

[10] Noctor G., Foyer C.H. (1998). Ascorbate and glutathione: Keeping active oxygen under control. Annu. Rev. Plant Physiol. Plant Mol. Biol..

[11] Caverzan A., Jardim-Messeder D., Paiva A.L., Margis-Pinheiro M., Panda S.K., Yamamoto Y. (2018). Ascorbate Peroxidases: Scavengers or Sensors of Hydrogen Peroxide Signaling?. Redox Homeostasis in Plants from Signalling to Stress Tolerance, Signaling and Communication in Plants.

[12] Teixeira F.K., Menezes-Benavente L., Galvao V.C., Margis R., Margis-Pinheiro M. (2006). Rice ascorbate peroxidase gene family encodes functionally diverse isoformslocalized in different subcellular compartments. Planta.

[13] Jardim-Messeder D., Caverzan A., Bastos G.A., Galhego V., Souza-Vieira Y., Lazzarotto F., Felix-Mendes E., Lavaquial L., Nicomedes Junior J., Margis-Pinheiro M. (2022). Genome-wide.; evolutionary.; and functional analyses of ascorbate peroxidase (APX) family in Poaceae species. Genet Mol. Biol..

[14] Caverzan A., Bonifacio A., Carvalho F.E.L., Andrade C.M.B., Passaia G., Schünemann M., Maraschin F.S., Martins M.O., Teixeira F.K., Rauber R. (2014). The knockdown of chloroplastic ascorbate peroxidases reveals its regulatory role in the photosynthesis and protection underphoto-oxidative stress in rice. Plant Sci..

[15] Jardim-Messeder D., Caverzan A., Rauber R., Cunha J.R., Carvalho F.E.L., Gaeta M.L., Fonseca G.C., Costa J.M., Frei M., Silveira J.A.G. (2018). Thylakoidal APX modulates hydrogen peroxide content and stomatal closure in rice (*Oryza sativa* L.). Environ. Exp. Bot..

[16] Cunha J.R., Carvalho F.E.L., Lima-Neto M.C., Jardim-Messeder D., Cerqueira J.V.A., Martins M.O., Fontenele A.V., Márgis-Pinheiro M., Komatsu S., Silveira J.A.G. (2019). Proteomic and physiological approaches reveal new insights for uncover the role of rice thylakoidal APX in response to drought stress. J. Proteomics..

[17] Jiang G., Yin D., Zhao J., Chen H., Guo L., Zhu L. (2016). The rice thylakoid membrane-bound ascorbate peroxidase OsAPX8 functions in tolerance to bacterial blight. Sci. Rep..

[18] Hoagland D.R., Arnon D.I. (1950). The Water-Culture Method for Growing Plants without Soil.

[19] Miki D., Shimamoto K. (2004). Simple RNAi vectors for stable and transient suppressionof gene function in rice. Plant Cell Physiol..

[20] Upadhyaya N.M., Zhou X.R., Zhu Q.H., Eamens A., Wang M.B., Water-house M.P., Dennis E.S., O’Brien L., Henry R.J. (2002). Transgenic Rice. Transgenic Cereals.

[21] Galván-Ampudia C.S., Offringa R. (2007). Plant evolution: AGC kinases tell the auxin tale. Trends Plant Sci..

[22] Chen S., Tao L., Zeng L., Vega-Sanchez M.E., Kenji Umemura W.G.L. (2006). A highly efficient transient protoplast system for analyzing defence gene expression and protein–protein interactions in rice. Mol. Plant Pathol..

[23] Tao L., Cheung A.Y., Wu H. (2002). Plant rac-like GTPases are activated by auxin and mediate auxin-responsive gene expression. Plant Cell.

[24] Jefferson R.A., Kavanagh T.A., Bevan M.W. (1987). GUS fusions: Beta-glucuronidase as a sensitive and versatile gene fusion marker in higher plants. EMBO J..

[25] Livak K.J., Schmittgen T.D. (2001). Analysis of relative gene expression data using real-time quantitative PCR and the 2^−ΔΔCT^ method. Methods.

[26] Schmittgen T.D., Livak K.J. (2008). Analyzing real-time PCR data by the comparative CT method. Nat. Protoc..

[27] Seigneurin-Berny D., Salvi D., Joyard J., Rolland N. (2008). Purification of intact chloroplasts from Arabidopsis and spinach leaves by isopycnic centrifugation. Curr. Protoc. Cell Biol..

[28] Rao M.V., Lee H., Creelman R.A., Mullet J.E., Davis K.R. (2000). Jasmonic acid signaling modulates ozone-induced hypersensitive cell death. Plant Cell.

[29] Smith A.M., Ratcliffe R.G., Sweetlove L.J. (2004). Activation and function of mitochondrial uncoupling protein in plants. J. Biol. Chem..

[30] Lichtenthaler H.K., Wellburn A.R. (1983). Determination of total carotenoids and chlorophylls a and b of leaf extracts in different solvents. Biochem. Soc. Trans..

[31] Blum A., Ebercon A. (1981). Cell membrane stability as a measure of drought and heat tolerance in wheat. Crop. Sci..

[32] Huang X.Y., Chao D.Y., Gao J.P., Zhu M.Z., Shi M., Lin H.X. (2009). A previously unknown zinc finger protein, DST, regulates drought and salt tolerance in rice via stomatal aperture control. Genes Dev..

[33] van Kooten O., Snel J.F. (1990). The use of chlorophyll fluorescence nomenclature in plant stress physiology. Photosynth. Res..

[34] Bilger W., Schreiber U., Bock M. (1995). Determination of the quantum efficiency of photosystem II and nonphotochemical quenching of chlorophyll fluorescence in the field. Oecologia.

[35] Bradford M.M. (1976). A rapid and sensitive for the quantitation of microgram quantitites of protein utilizing the principle of protein-dye binding. Anal. Biochem..

[36] Koshiba T. (1993). Cytosolic ascorbate peroxidase in seedlings and leaves of maize (Zea mays). Plant Cell Physiol..

[37] Andrews S. (2010). FastQC: A Quality Control Tool for High Throughput Sequence Data. https://www.bibsonomy.org/bibtex/2b6052877491828ab53d3449be9b293b3/ozborn.

[38] Kim D., Paggi J.M., Park C., Bennet C., Salzberg S.L. (2019). Graph-based genome alignment and genotyping with HISAT2 and HISAT-genotype. Nat Biotechnol..

[39] Varet H., Brillet-Guéguen L., Coppee J.Y., Dillies M.A. (2016). SARTools: A DESeq2- and EdgeR-Based R Pipeline for Comprehensive Differential Analysis of RNA-Seq Data. PLoS ONE.

[40] Benjamini Y., Hochberg Y. (1995). Controlling the false discovery rate: A practical and powerful approach to multiple testing. J. R. Stat. Soc. Ser. B (Methodol.).

[41] Ge S.X., Jung D., Yao R. (2020). ShinyGO: A graphical gene-set enrichment tool for animals and plants. Bioinformatics.

[42] Hooper S.D., Bork P. (2005). Medusa: A simple tool for interaction graph analysis. Bioinformatics.

[43] Castro M.A., Filho J.L., Dalmolin R.J., Sinigaglia M., Moreira J.C., Mombach J.C., de Almeida R.M. (2009). ViaComplex: Software for landscape analysis of gene expression networks in genomic context. Bioinformatics.

[44] Jardim-Messeder D., Zamocky M., Sachetto-Martins G., Margis-Pinheiro M. (2022). Chloroplastic ascorbate peroxidases targeted to stroma or thylakoid membrane: The chicken or egg dilemma. FEBS Lett..

[45] Van Camp W., Capiau K., Van Montagu M., Inze D., Slooten L. (1996). Enhancement of oxidative stress tolerance in transgenic tobacco plants overproducing Fe-superoxide dismutase in chloroplasts. Plant Physiol..

[46] Hernandez J.A., Ferrer M.A., Jiménez A., Barcelo A.R., Sevilla F. (2001). Antioxidant systems and O_2_^-^/H_2_O_2_ production in the apoplast of pea leaves. Its relation with salt-induced necrotic lesions in minor veins. Plant Physiol..

[47] Mittler R., Berkowitz G. (2001). Hydrogen peroxide.; a messenger with too many roles?. Redox Rep..

[48] Tseng M.J., Liu C.W., Yiu J.C. (2017). Enhanced tolerance to sulfur dioxide and salt stress of transgenic Chinese cabbage plants expressing both superoxide dismutase and catalase in chloroplasts. Plant Physiol. Biochem..

[49] Daszkowska-Golec A., Szarejko I. (2013). Open or Close the Gate–Stomata Action Under the Control of Phytohormones in Drought Stress Conditions. Front Plant Sci..

[50] Jones H.G. (1999). Use of thermography for quantitative studies of spatial and temporal variation of stomatal conductance over leaf surfaces. Plant Cell Environ..

[51] Sirault X.R.R., James R.A., Furbank R.T. (2009). A new screening method for osmotic component of salinity tolerance in cereals using infrared thermography. Funct. Plant Biol..

[52] Cao M.C., Zhang W.Z., Han Y.D., Yao C., Wang Y.T., Ding G.H. (2013). A theoretical model research of rice water stress index based on automated infrared thermal imaging. Adv. Mater. Res..

[53] Carvalho F.E.L., Ribeiro C.W., Martins M.O., Bonifacio A., Staats C.C., Andrade C.M., Cerqueira J.V., Margis-Pinheiro M., Silveira J.A.G. (2014). Cytosolic APX knockdown rice plants sustain photosynthesis by regulation of protein expression related to photochemistry, Calvin cycle and photorespiration. Physiol. Plant..

[54] Xu F.Q., Xue H.W. (2019). The ubiquitin-proteasome system in plant responses to environments. Plant Cell Environ..

[55] Ali M.S., Baek K.H. (2020). Protective roles of cytosolic and plastidal proteasomes on abiotic stress and pathogen invasion. Plants.

[56] Kurepa J., Toh-E A., Smalle J.A. (2008). 26S proteasome regulatory particle mutants have increased oxidative stress tolerance. Plant J..

[57] Hoshida H., Tanaka Y., Hibino T., Hayashi Y., Tanaka A., Takabe T., Takabe T. (2000). Enhanced tolerance to salt stress in transgenic rice that overexpresses chloroplast glutamine synthetase. Plant Mol. Biol..

[58] Gao Z., Wang Y., Chen G., Zhang A., Yang S., Shang L., Wang D., Ruan B., Liu C., Jiang H. (2019). The indica nitrate reductase gene OsNR2 allele enhances rice yield potential and nitrogen use efficiency. Nat Commun..

[59] Polishchuk O. (2021). Stress-Related Changes in the Expression and Activity of Plant Carbonic Anhydrases. Planta.

[60] Wang X., Li B., Ma T.T., Sun L.Y., Tai L., Hu C.H., Liu W.T., Li W.Q., Chen K.M. (2020). The NAD kinase OsNADK1 affects the intracellular redox balance and enhances the tolerance of rice to drought. BMC Plant Biol..

[61] Wang H., Dong Q., Duan D., Zhao S., Li M., van Nocker S., Ma F., Mao K. (2018). Comprehensive genomic analysis of the TYROSINE AMINOTRANSFERASE (TAT) genes in apple (*Malus domestica*) allows the identification of MdTAT2 conferring tolerance to drought and osmotic stresses in plants. Plant Physiol. Biochem..

[62] Kumar V.V.S., Yadav S.K., Verma R.K., Shrivastava S., Ghimire O., Pushkar S., Rao M.V., Kumar T.S., Chinnusamy V. (2021). The abscisic acid receptor OsPYL6 confers drought tolerance to indica rice through dehydration avoidance and tolerance mechanisms. J. Exp. Bot..

