# Supplementary material for: Stromal Ascorbate Peroxidase (OsAPX7) Modulates Drought Stress Tolerance in Rice (Oryza sativa)
Source: Antioxidants (Basel). 2023 Feb 5;12(2):387. doi: 10.3390/antiox12020387 (PMC9952370; doi:10.3390/antiox12020387)
Supplement: Supplementary file 1 [file antioxidants-12-00387-s001.zip › Supplementary Figures - Antioxidants - Jardim-Messeder et al 30-12-22.pptx]

## Slide 1
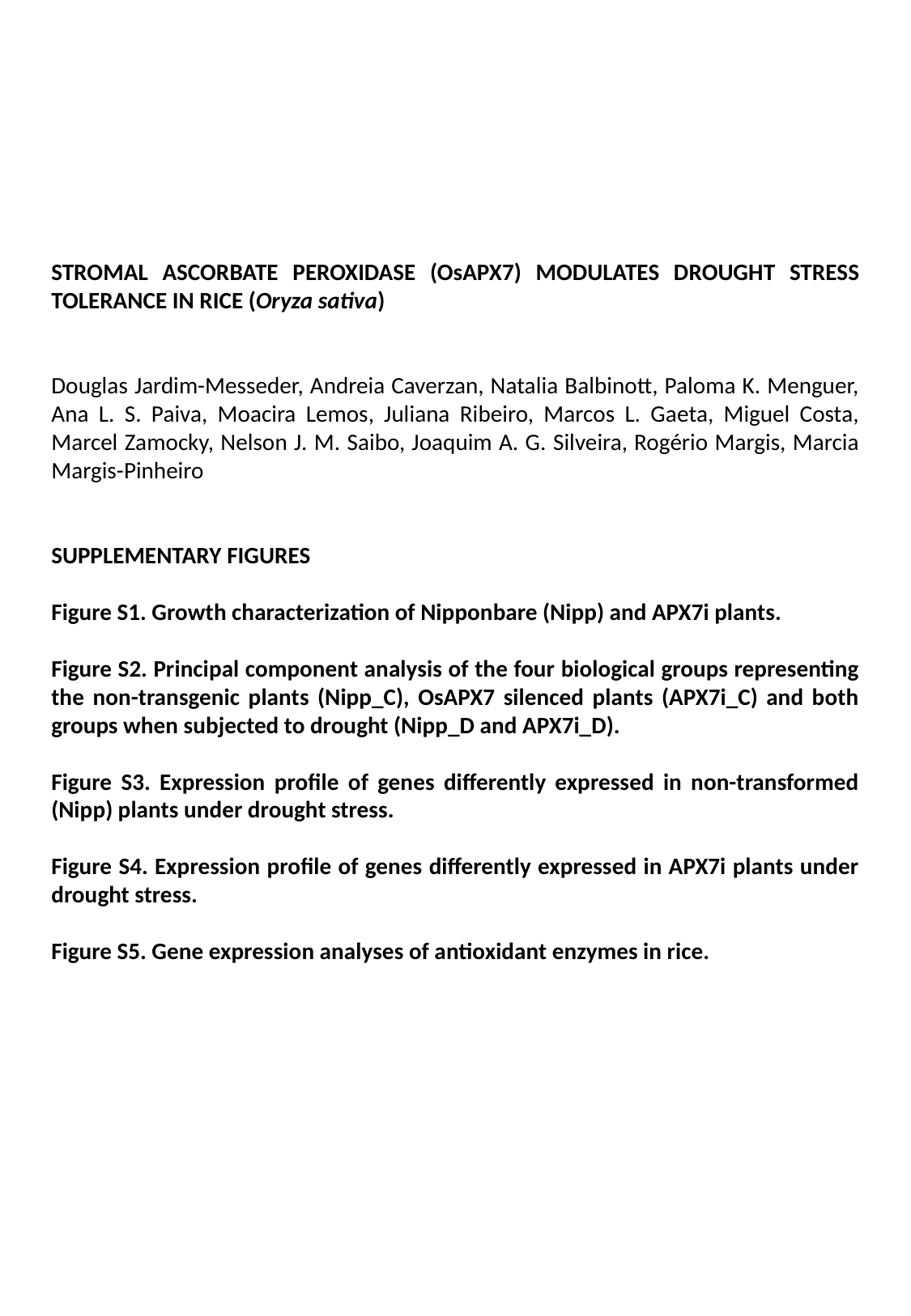

STROMAL ASCORBATE PEROXIDASE (OsAPX7) MODULATES DROUGHT STRESS TOLERANCE IN RICE (Oryza sativa)
Douglas Jardim-Messeder, Andreia Caverzan, Natalia Balbinott, Paloma K. Menguer, Ana L. S. Paiva, Moacira Lemos, Juliana Ribeiro, Marcos L. Gaeta, Miguel Costa, Marcel Zamocky, Nelson J. M. Saibo, Joaquim A. G. Silveira, Rogério Margis, Marcia Margis-Pinheiro
SUPPLEMENTARY FIGURES
Figure S1. Growth characterization of Nipponbare (Nipp) and APX7i plants.
Figure S2. Principal component analysis of the four biological groups representing the non-transgenic plants (Nipp_C), OsAPX7 silenced plants (APX7i_C) and both groups when subjected to drought (Nipp_D and APX7i_D).
Figure S3. Expression profile of genes differently expressed in non-transformed (Nipp) plants under drought stress.
Figure S4. Expression profile of genes differently expressed in APX7i plants under drought stress.
Figure S5. Gene expression analyses of antioxidant enzymes in rice.

## Slide 2
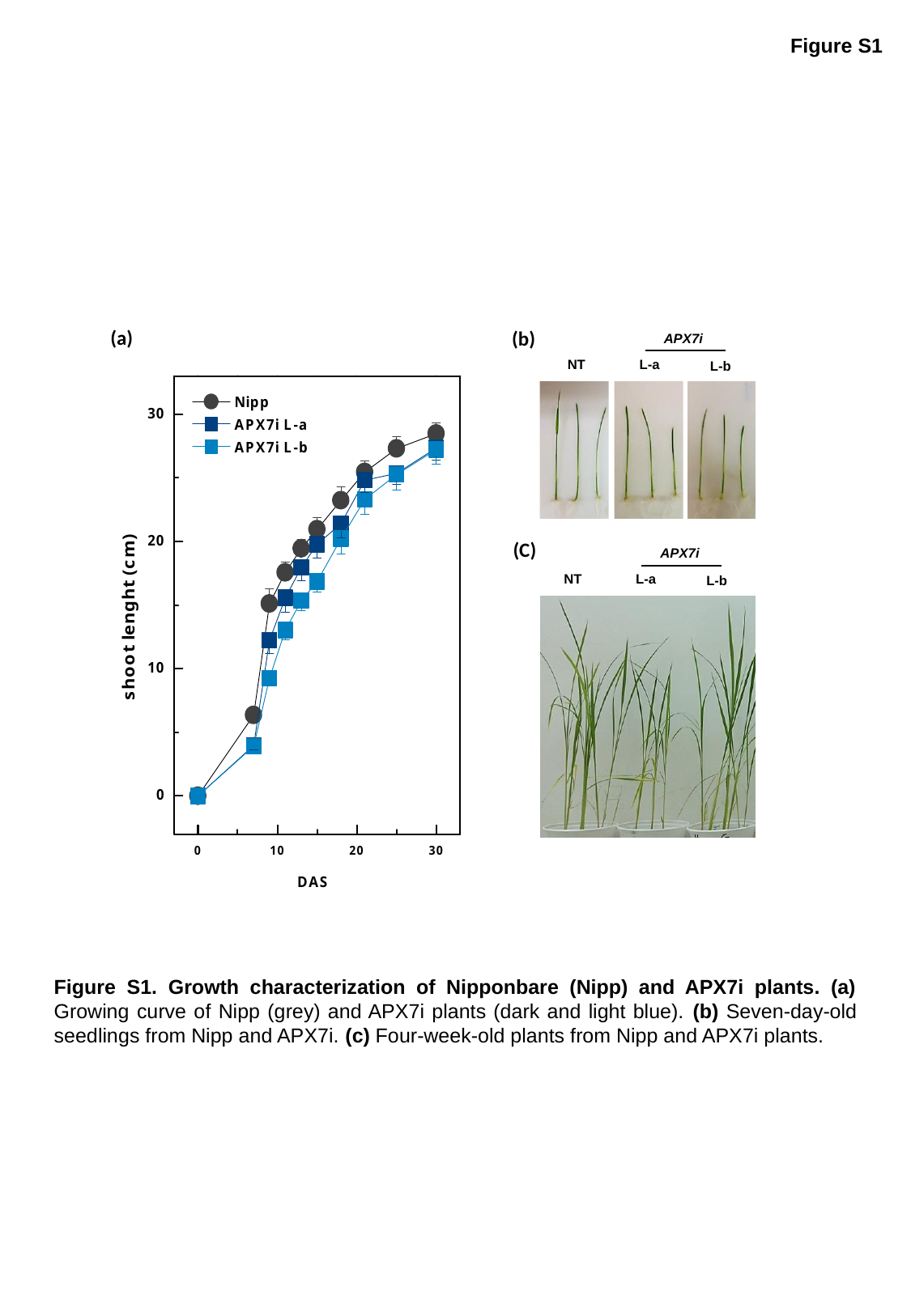

Figure S1
(a)
(b)
APX7i
NT
L-a
L-b
(C)
APX7i
NT
L-a
L-b
Figure S1. Growth characterization of Nipponbare (Nipp) and APX7i plants. (a) Growing curve of Nipp (grey) and APX7i plants (dark and light blue). (b) Seven-day-old seedlings from Nipp and APX7i. (c) Four-week-old plants from Nipp and APX7i plants.

## Slide 3
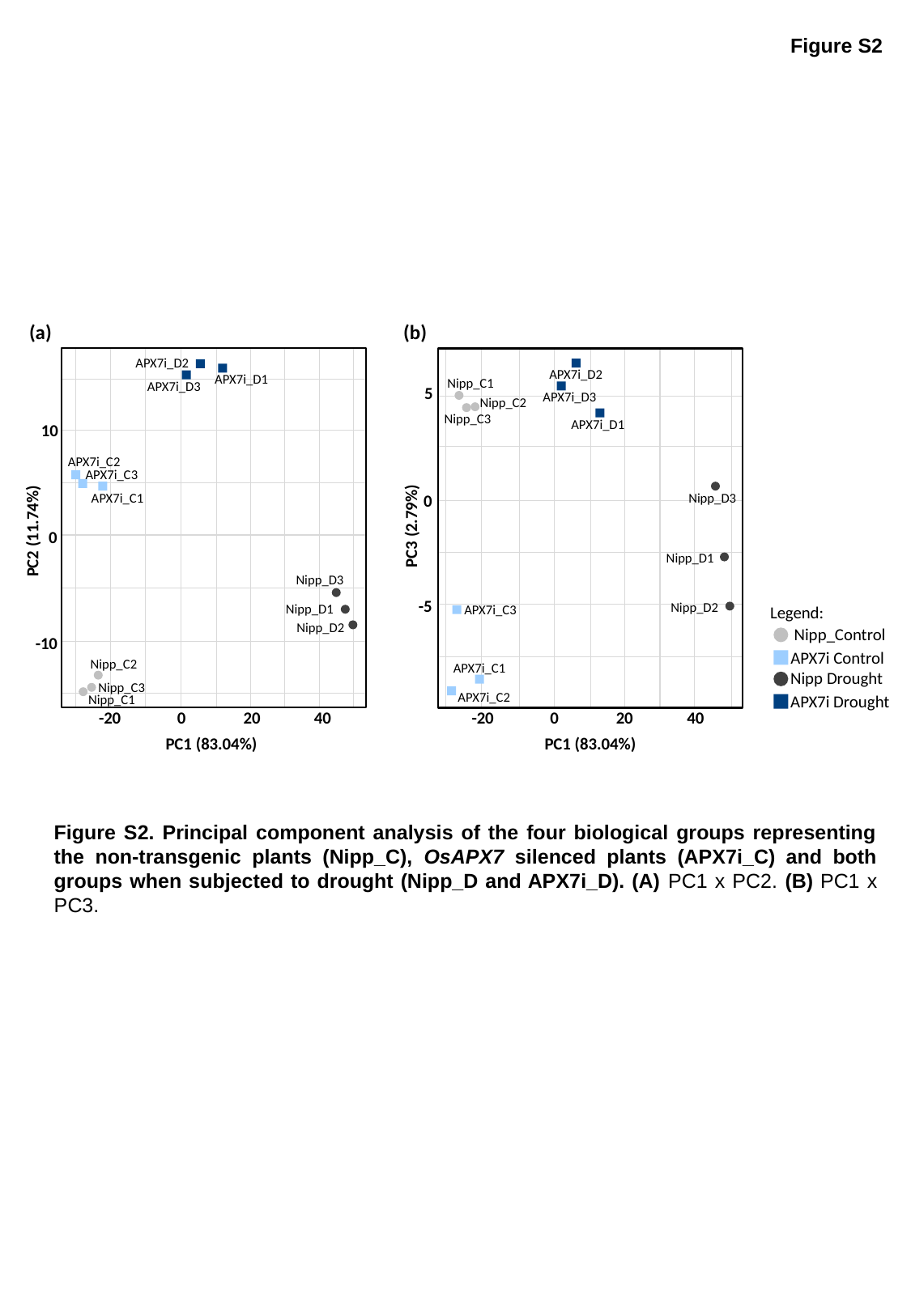

Figure S2
(a)
APX7i_D2
APX7i_D1
APX7i_D3
10
APX7i_C2
APX7i_C3
APX7i_C1
PC2 (11.74%)
0
Nipp_D3
Nipp_D1
Nipp_D2
-10
Nipp_C2
Nipp_C3
Nipp_C1
-20
0
20
40
PC1 (83.04%)
(b)
APX7i_D2
Nipp_C1
5
APX7i_D3
Nipp_C2
Nipp_C3
APX7i_D1
Nipp_D3
0
PC3 (2.79%)
Nipp_D1
-5
Nipp_D2
APX7i_C3
APX7i_C1
APX7i_C2
-20
0
20
40
PC1 (83.04%)
Legend:
Nipp_Control
APX7i Control
Nipp Drought
APX7i Drought
Figure S2. Principal component analysis of the four biological groups representing the non-transgenic plants (Nipp_C), OsAPX7 silenced plants (APX7i_C) and both groups when subjected to drought (Nipp_D and APX7i_D). (A) PC1 x PC2. (B) PC1 x PC3.

## Slide 4
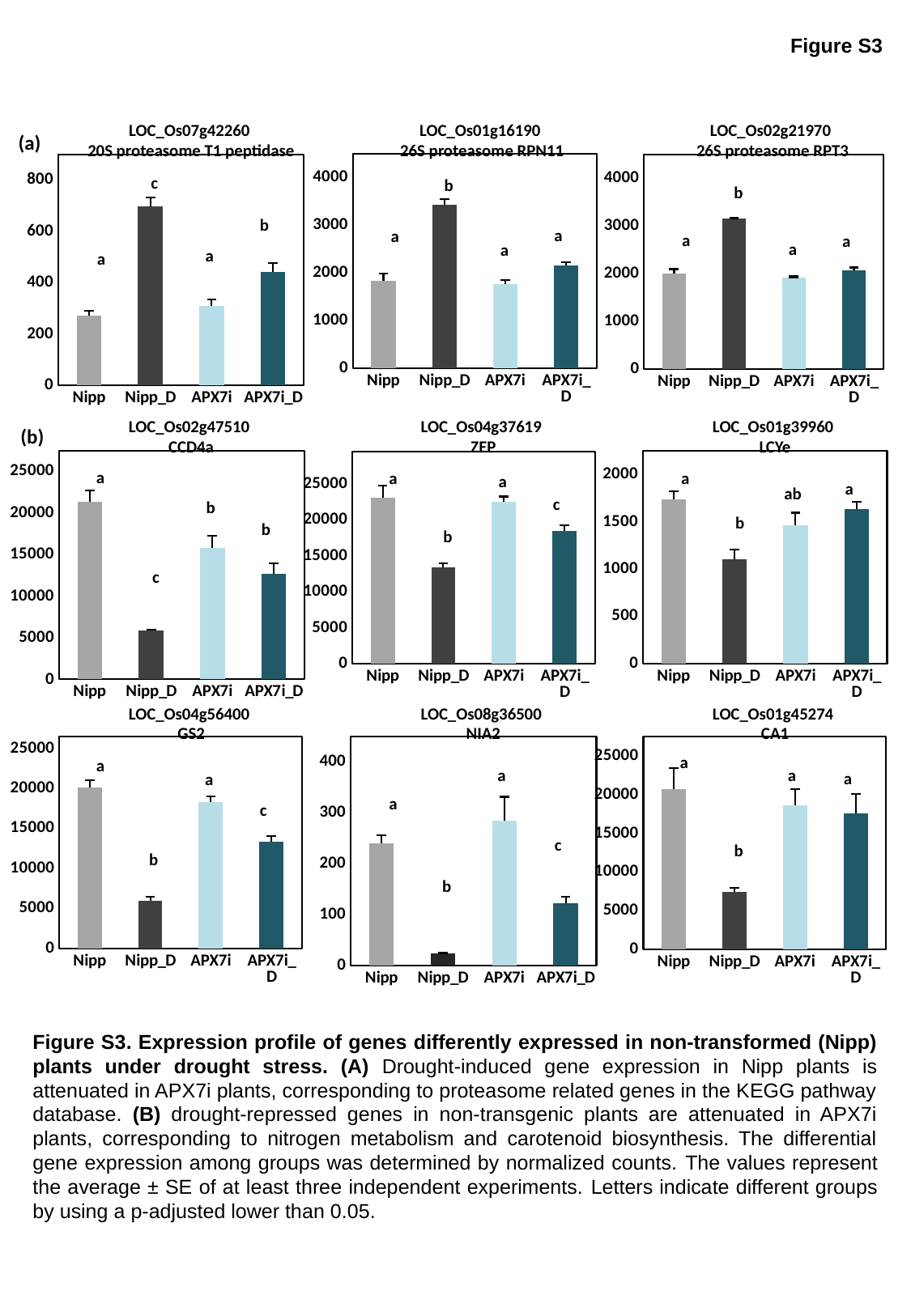

Figure S3
LOC_Os07g42260
 20S proteasome T1 peptidase
LOC_Os01g16190
 26S proteasome RPN11
LOC_Os02g21970
 26S proteasome RPT3
(a)
### Chart
| Category | |
|---|---|
| Nipp | 1821.3333333333333 |
| Nipp_D | 3429.0 |
| APX7i | 1755.3333333333333 |
| APX7i_D | 2148.6666666666665 |
### Chart
| Category | |
|---|---|
| Nipp | 2008.3333333333333 |
| Nipp_D | 3153.3333333333335 |
| APX7i | 1925.3333333333333 |
| APX7i_D | 2078.0 |
### Chart
| Category | |
|---|---|
| Nipp | 273.0 |
| Nipp_D | 697.0 |
| APX7i | 308.3333333333333 |
| APX7i_D | 443.6666666666667 |
### Chart
| Category | |
|---|---|
| Nipp | 21355.333333333332 |
| Nipp_D | 5913.0 |
| APX7i | 15838.0 |
| APX7i_D | 12721.666666666666 |
### Chart
| Category | |
|---|---|
| Nipp | 1740.3333333333333 |
| Nipp_D | 1102.3333333333333 |
| APX7i | 1465.6666666666667 |
| APX7i_D | 1633.0 |
### Chart
| Category | |
|---|---|
| Nipp | 23109.666666666668 |
| Nipp_D | 13435.333333333334 |
| APX7i | 22457.333333333332 |
| APX7i_D | 18437.0 |
### Chart
| Category | |
|---|---|
| Nipp | 20086.333333333332 |
| Nipp_D | 5979.0 |
| APX7i | 18326.666666666668 |
| APX7i_D | 13288.0 |
### Chart
| Category | |
|---|---|
| Nipp | 240.33333333333334 |
| Nipp_D | 23.0 |
| APX7i | 283.6666666666667 |
| APX7i_D | 122.0 |
### Chart
| Category | |
|---|---|
| Nipp | 20735.333333333332 |
| Nipp_D | 7418.0 |
| APX7i | 18621.333333333332 |
| APX7i_D | 17608.333333333332 |(b)
LOC_Os02g47510
 CCD4a
LOC_Os04g37619
 ZEP
LOC_Os01g39960
 LCYe
LOC_Os04g56400
 GS2
LOC_Os08g36500
 NIA2
LOC_Os01g45274
 CA1
c
b
b
b
a
a
a
a
a
a
a
a
a
a
a
a
a
ab
c
b
b
b
b
c
a
a
a
a
a
a
a
c
c
b
b
b
Figure S3. Expression profile of genes differently expressed in non-transformed (Nipp) plants under drought stress. (A) Drought-induced gene expression in Nipp plants is attenuated in APX7i plants, corresponding to proteasome related genes in the KEGG pathway database. (B) drought-repressed genes in non-transgenic plants are attenuated in APX7i plants, corresponding to nitrogen metabolism and carotenoid biosynthesis. The differential gene expression among groups was determined by normalized counts. The values represent the average ± SE of at least three independent experiments. Letters indicate different groups by using a p-adjusted lower than 0.05.

## Slide 5
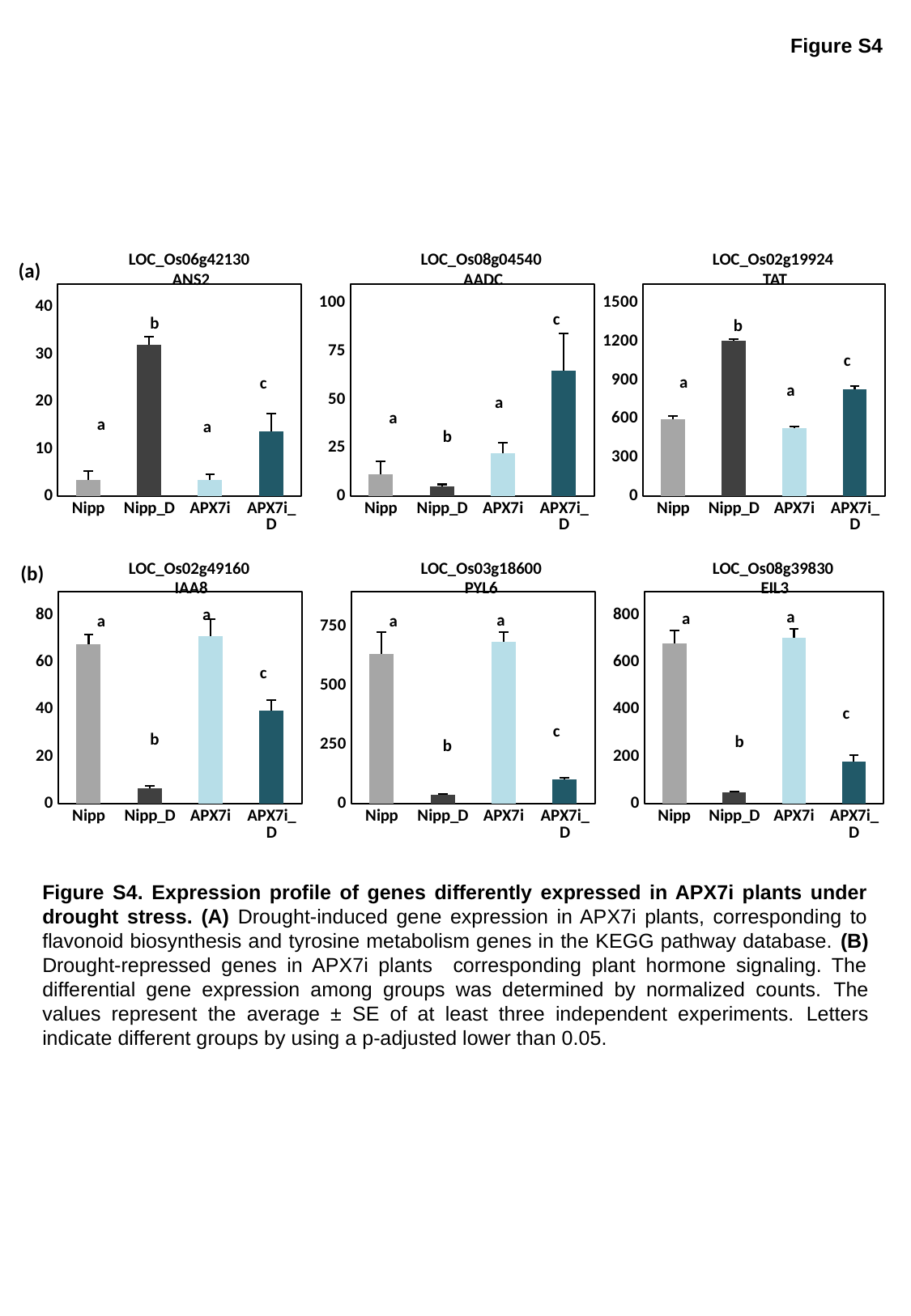

Figure S4
LOC_Os06g42130
 ANS2
LOC_Os08g04540
 AADC
LOC_Os02g19924
 TAT
(a)
### Chart
| Category | |
|---|---|
| Nipp | 3.3333333333333335 |
| Nipp_D | 32.0 |
| APX7i | 3.3333333333333335 |
| APX7i_D | 13.666666666666666 |
### Chart
| Category | |
|---|---|
| Nipp | 11.333333333333334 |
| Nipp_D | 5.0 |
| APX7i | 22.333333333333332 |
| APX7i_D | 65.0 |
### Chart
| Category | |
|---|---|
| Nipp | 596.6666666666666 |
| Nipp_D | 1205.6666666666667 |
| APX7i | 529.3333333333334 |
| APX7i_D | 829.3333333333334 |
### Chart
| Category | |
|---|---|
| Nipp | 67.66666666666667 |
| Nipp_D | 6.666666666666667 |
| APX7i | 71.0 |
| APX7i_D | 39.333333333333336 |
### Chart
| Category | |
|---|---|
| Nipp | 636.3333333333334 |
| Nipp_D | 39.0 |
| APX7i | 685.3333333333334 |
| APX7i_D | 103.33333333333333 |
### Chart
| Category | |
|---|---|
| Nipp | 679.3333333333334 |
| Nipp_D | 48.666666666666664 |
| APX7i | 703.0 |
| APX7i_D | 177.66666666666666 |LOC_Os02g49160
 IAA8
LOC_Os03g18600
PYL6
LOC_Os08g39830
 EIL3
(b)
c
b
b
c
a
c
a
a
a
a
a
b
a
a
a
a
a
a
c
c
c
b
b
b
Figure S4. Expression profile of genes differently expressed in APX7i plants under drought stress. (A) Drought-induced gene expression in APX7i plants, corresponding to flavonoid biosynthesis and tyrosine metabolism genes in the KEGG pathway database. (B) Drought-repressed genes in APX7i plants corresponding plant hormone signaling. The differential gene expression among groups was determined by normalized counts. The values represent the average ± SE of at least three independent experiments. Letters indicate different groups by using a p-adjusted lower than 0.05.

## Slide 6
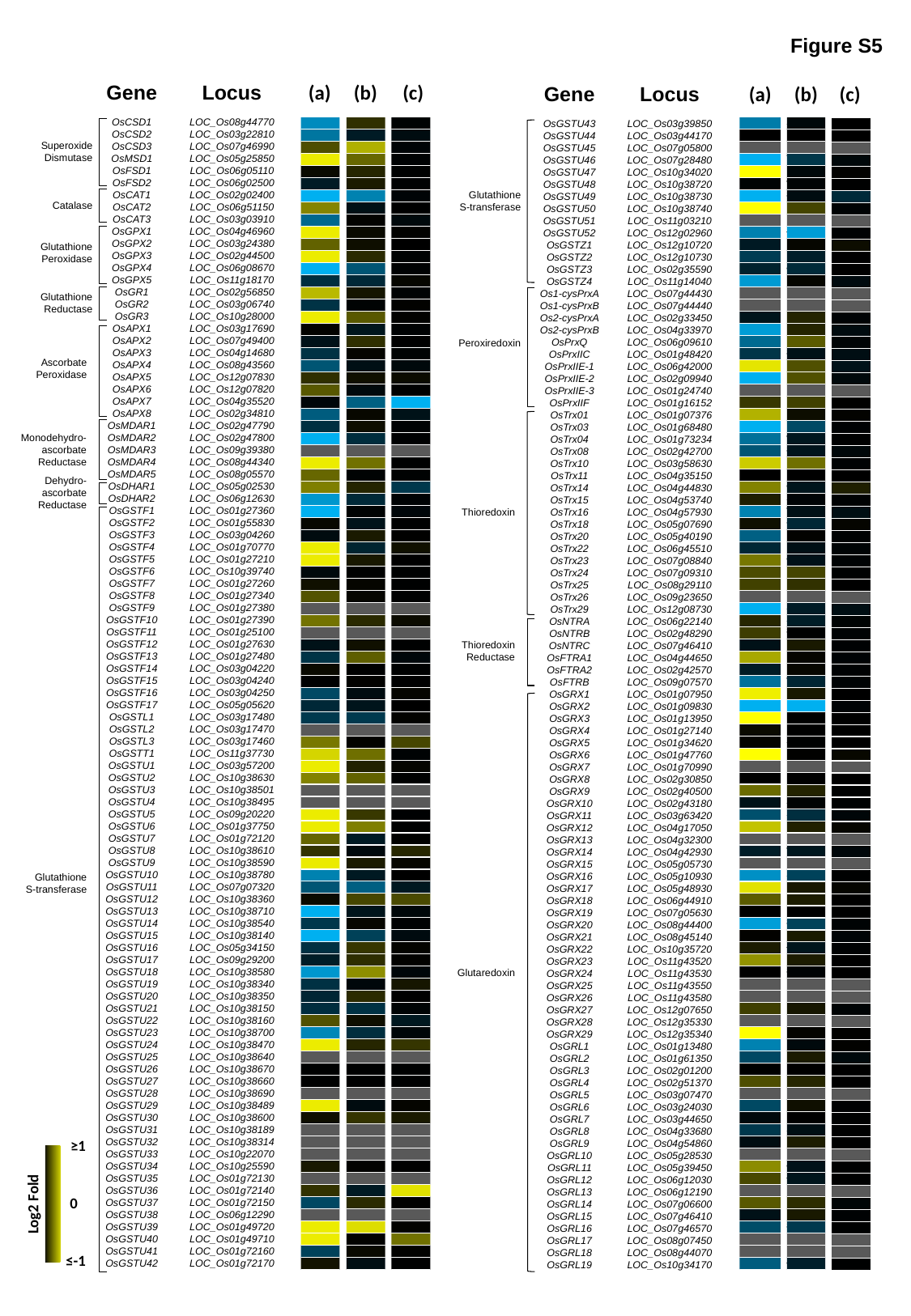

Figure S5
Gene
OsCSD1
OsCSD2
OsCSD3
OsMSD1
OsFSD1
OsFSD2
OsCAT1
OsCAT2
OsCAT3
OsGPX1
OsGPX2
OsGPX3
OsGPX4
OsGPX5
OsGR1
OsGR2
OsGR3
OsAPX1
OsAPX2
OsAPX3
OsAPX4
OsAPX5
OsAPX6
OsAPX7
OsAPX8
OsMDAR1
OsMDAR2
OsMDAR3
OsMDAR4
OsMDAR5
OsDHAR1
OsDHAR2
OsGSTF1
OsGSTF2
OsGSTF3
OsGSTF4
OsGSTF5
OsGSTF6
OsGSTF7
OsGSTF8
OsGSTF9
OsGSTF10
OsGSTF11
OsGSTF12
OsGSTF13
OsGSTF14
OsGSTF15
OsGSTF16
OsGSTF17
OsGSTL1
OsGSTL2
OsGSTL3
OsGSTT1
OsGSTU1
OsGSTU2
OsGSTU3
OsGSTU4
OsGSTU5
OsGSTU6
OsGSTU7
OsGSTU8
OsGSTU9
OsGSTU10
OsGSTU11
OsGSTU12
OsGSTU13
OsGSTU14
OsGSTU15
OsGSTU16
OsGSTU17
OsGSTU18
OsGSTU19
OsGSTU20
OsGSTU21
OsGSTU22
OsGSTU23
OsGSTU24
OsGSTU25
OsGSTU26
OsGSTU27
OsGSTU28
OsGSTU29
OsGSTU30
OsGSTU31
OsGSTU32
OsGSTU33
OsGSTU34
OsGSTU35
OsGSTU36
OsGSTU37
OsGSTU38
OsGSTU39
OsGSTU40
OsGSTU41
OsGSTU42
Locus
LOC_Os08g44770
LOC_Os03g22810
LOC_Os07g46990
LOC_Os05g25850
LOC_Os06g05110
LOC_Os06g02500
LOC_Os02g02400
LOC_Os06g51150
LOC_Os03g03910
LOC_Os04g46960
LOC_Os03g24380
LOC_Os02g44500
LOC_Os06g08670
LOC_Os11g18170
LOC_Os02g56850
LOC_Os03g06740
LOC_Os10g28000
LOC_Os03g17690
LOC_Os07g49400
LOC_Os04g14680
LOC_Os08g43560
LOC_Os12g07830
LOC_Os12g07820
LOC_Os04g35520
LOC_Os02g34810
LOC_Os02g47790
LOC_Os02g47800
LOC_Os09g39380
LOC_Os08g44340
LOC_Os08g05570
LOC_Os05g02530
LOC_Os06g12630
LOC_Os01g27360
LOC_Os01g55830
LOC_Os03g04260
LOC_Os01g70770
LOC_Os01g27210
LOC_Os10g39740
LOC_Os01g27260
LOC_Os01g27340
LOC_Os01g27380
LOC_Os01g27390
LOC_Os01g25100
LOC_Os01g27630
LOC_Os01g27480
LOC_Os03g04220
LOC_Os03g04240
LOC_Os03g04250
LOC_Os05g05620
LOC_Os03g17480
LOC_Os03g17470
LOC_Os03g17460
LOC_Os11g37730
LOC_Os03g57200
LOC_Os10g38630
LOC_Os10g38501
LOC_Os10g38495
LOC_Os09g20220
LOC_Os01g37750
LOC_Os01g72120
LOC_Os10g38610
LOC_Os10g38590
LOC_Os10g38780
LOC_Os07g07320
LOC_Os10g38360
LOC_Os10g38710
LOC_Os10g38540
LOC_Os10g38140
LOC_Os05g34150
LOC_Os09g29200
LOC_Os10g38580
LOC_Os10g38340
LOC_Os10g38350
LOC_Os10g38150
LOC_Os10g38160
LOC_Os10g38700
LOC_Os10g38470
LOC_Os10g38640
LOC_Os10g38670
LOC_Os10g38660
LOC_Os10g38690
LOC_Os10g38489
LOC_Os10g38600
LOC_Os10g38189
LOC_Os10g38314
LOC_Os10g22070
LOC_Os10g25590
LOC_Os01g72130
LOC_Os01g72140
LOC_Os01g72150
LOC_Os06g12290
LOC_Os01g49720
LOC_Os01g49710
LOC_Os01g72160
LOC_Os01g72170
(a)
(b)
(c)
Gene
OsGSTU43
OsGSTU44
OsGSTU45
OsGSTU46
OsGSTU47
OsGSTU48
OsGSTU49
OsGSTU50
OsGSTU51
OsGSTU52
OsGSTZ1
OsGSTZ2
OsGSTZ3
OsGSTZ4
Os1-cysPrxA
Os1-cysPrxB
Os2-cysPrxA
Os2-cysPrxB
OsPrxQ
OsPrxIIC
OsPrxIIE-1
OsPrxIIE-2
OsPrxIIE-3
OsPrxIIF
OsTrx01
OsTrx03
OsTrx04
OsTrx08
OsTrx10
OsTrx11
OsTrx14
OsTrx15
OsTrx16
OsTrx18
OsTrx20
OsTrx22
OsTrx23
OsTrx24
OsTrx25
OsTrx26
OsTrx29
OsNTRA
OsNTRB
OsNTRC
OsFTRA1
OsFTRA2
OsFTRB
OsGRX1
OsGRX2
OsGRX3
OsGRX4
OsGRX5
OsGRX6
OsGRX7
OsGRX8
OsGRX9
OsGRX10
OsGRX11
OsGRX12
OsGRX13
OsGRX14
OsGRX15
OsGRX16
OsGRX17
OsGRX18
OsGRX19
OsGRX20
OsGRX21
OsGRX22
OsGRX23
OsGRX24
OsGRX25
OsGRX26
OsGRX27
OsGRX28
OsGRX29
OsGRL1
OsGRL2
OsGRL3
OsGRL4
OsGRL5
OsGRL6
OsGRL7
OsGRL8
OsGRL9
OsGRL10
OsGRL11
OsGRL12
OsGRL13
OsGRL14
OsGRL15
OsGRL16
OsGRL17
OsGRL18
OsGRL19
Locus
LOC_Os03g39850
LOC_Os03g44170
LOC_Os07g05800
LOC_Os07g28480
LOC_Os10g34020
LOC_Os10g38720
LOC_Os10g38730
LOC_Os10g38740
LOC_Os11g03210
LOC_Os12g02960
LOC_Os12g10720
LOC_Os12g10730
LOC_Os02g35590
LOC_Os11g14040
LOC_Os07g44430
LOC_Os07g44440
LOC_Os02g33450
LOC_Os04g33970
LOC_Os06g09610
LOC_Os01g48420
LOC_Os06g42000
LOC_Os02g09940
LOC_Os01g24740
LOC_Os01g16152
LOC_Os01g07376
LOC_Os01g68480
LOC_Os01g73234
LOC_Os02g42700
LOC_Os03g58630
LOC_Os04g35150
LOC_Os04g44830
LOC_Os04g53740
LOC_Os04g57930
LOC_Os05g07690
LOC_Os05g40190
LOC_Os06g45510
LOC_Os07g08840
LOC_Os07g09310
LOC_Os08g29110
LOC_Os09g23650
LOC_Os12g08730
LOC_Os06g22140
LOC_Os02g48290
LOC_Os07g46410
LOC_Os04g44650
LOC_Os02g42570
LOC_Os09g07570
LOC_Os01g07950
LOC_Os01g09830
LOC_Os01g13950
LOC_Os01g27140
LOC_Os01g34620
LOC_Os01g47760
LOC_Os01g70990
LOC_Os02g30850
LOC_Os02g40500
LOC_Os02g43180
LOC_Os03g63420
LOC_Os04g17050
LOC_Os04g32300
LOC_Os04g42930
LOC_Os05g05730
LOC_Os05g10930
LOC_Os05g48930
LOC_Os06g44910
LOC_Os07g05630
LOC_Os08g44400
LOC_Os08g45140
LOC_Os10g35720
LOC_Os11g43520
LOC_Os11g43530
LOC_Os11g43550
LOC_Os11g43580
LOC_Os12g07650
LOC_Os12g35330
LOC_Os12g35340
LOC_Os01g13480
LOC_Os01g61350
LOC_Os02g01200
LOC_Os02g51370
LOC_Os03g07470
LOC_Os03g24030
LOC_Os03g44650
LOC_Os04g33680
LOC_Os04g54860
LOC_Os05g28530
LOC_Os05g39450
LOC_Os06g12030
LOC_Os06g12190
LOC_Os07g06600
LOC_Os07g46410
LOC_Os07g46570
LOC_Os08g07450
LOC_Os08g44070
LOC_Os10g34170
(a)
(b)
(c)
Superoxide
Dismutase
Glutathione
S-transferase
Catalase
Glutathione
Peroxidase
Glutathione
Reductase
Peroxiredoxin
Ascorbate
Peroxidase
Monodehydro-
ascorbate
Reductase
Dehydro-
ascorbate
Reductase
Thioredoxin
Thioredoxin
Reductase
Glutathione
S-transferase
Glutaredoxin
≥1
Log2 Fold
0
≤-1

## Slide 7
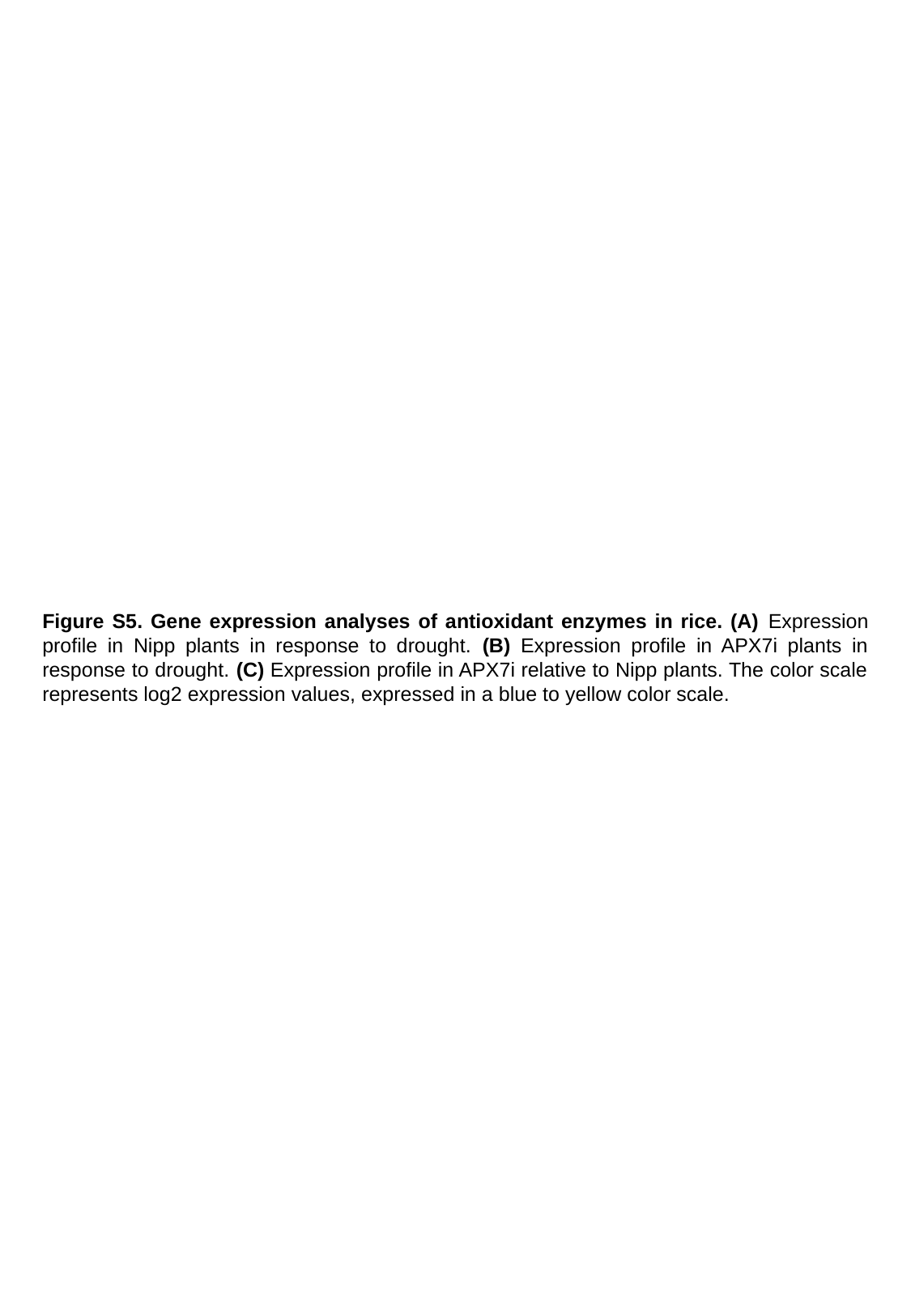

Figure S5. Gene expression analyses of antioxidant enzymes in rice. (A) Expression profile in Nipp plants in response to drought. (B) Expression profile in APX7i plants in response to drought. (C) Expression profile in APX7i relative to Nipp plants. The color scale represents log2 expression values, expressed in a blue to yellow color scale.
